# Linear spline multilevel models for summarising childhood growth trajectories: A guide to their application using examples from five birth cohorts

**DOI:** 10.1177/0962280213503925

**Published:** 2013-10-09

**Authors:** Laura D Howe, Kate Tilling, Alicia Matijasevich, Emily S Petherick, Ana Cristina Santos, Lesley Fairley, John Wright, Iná S. Santos, Aluísio JD Barros, Richard M Martin, Michael S Kramer, Natalia Bogdanovich, Lidia Matush, Henrique Barros, Debbie A Lawlor

**Affiliations:** 1 MRC Integrative Epidemiology Unit at the University of Bristol, School of Social and Community Medicine, University of Bristol, UK; 2 School of Social and Community Medicine, University of Bristol, UK; 3 Postgraduate Programme in Epidemiology, Federal University of Pelotas, Pelotas, Brazil; 4 Bradford Teaching Hospitals NHS Foundation Trust, Bradford, UK; 5 Department of Clinical Epidemiology, Predictive Medicine and Public Health, University of Porto Medical School, University of Porto Institute of Public Health, Porto, Portugal; 6 National Institute for Health Research Bristol Nutrition Biomedical Research Unit, University of Bristol / University Hospitals Bristol NHS Foundation Trust, Bristol, UK; 7 Department of Pediatrics, Faculty of Medicine, McGill University, Montreal, Canada; 8 Belarusian Ministry of Health and Belarussian Maternal and Child Health Research Institute, Minsk*,* Belarus

**Keywords:** child, growth, height, longitudinal, multilevel models, spline, weight, ALSPAC, Born in Bradford, Generation XXI, Pelotas, PROBIT

## Abstract

Childhood growth is of interest in medical research concerned with determinants and consequences of variation from healthy growth and development. Linear spline multilevel modelling is a useful approach for deriving individual summary measures of growth, which overcomes several data issues (co-linearity of repeat measures, the requirement for all individuals to be measured at the same ages and bias due to missing data). Here, we outline the application of this methodology to model individual trajectories of length/height and weight, drawing on examples from five cohorts from different generations and different geographical regions with varying levels of economic development. We describe the unique features of the data within each cohort that have implications for the application of linear spline multilevel models, for example, differences in the density and inter-individual variation in measurement occasions, and multiple sources of measurement with varying measurement error. After providing example Stata syntax and a suggested workflow for the implementation of linear spline multilevel models, we conclude with a discussion of the advantages and disadvantages of the linear spline approach compared with other growth modelling methods such as fractional polynomials, more complex spline functions and other non-linear models.

## 1 Introduction

### 1.1 Background

Childhood growth is a key indicator of a child’s health and development,^1^ and is also an important influence on health and wellbeing later in life.^2–5^

There is evidence that the growth patterns of children, in the absence of ill-health or other adverse environmental conditions, are remarkably similar across different countries of the world, even those with very different levels of economic development.^6^ However, poor health and nutrition can prevent a child from attaining their genetically determined growth potential.^1^ The geographical and social patterning of these health and nutritional insults results in growth differences between high-, middle- and low-income countries,^7^ as well as generating within-country socioeconomic differentials in growth and final body size.^8,9^

Appropriate modelling of longitudinal data is required in order to compare growth patterns across populations, examine factors that influence growth or assess associations between growth and later outcomes. This presents several statistical challenges. The non-independence of repeat measures within an individual must be considered in order to obtain appropriate standard errors. Traditional methods that have been used in the analysis of child growth data, such as the inclusion of z-scores for multiple child growth measures in a multiple regression model,^10^ do not properly model the clustering of measurements within individuals. As children grow, the scale of growth measurements, and therefore variance and measurement error, increases with age. Although z-score-based methods address this by standardising the measurements across time, they do not allow the true shape of growth trajectories to be modelled. Sampling designs within many studies also present challenges for the analysis of growth data. For example, there may not be balance in the timing of growth measurements available for each individual. Even when follow-ups are planned for certain ages, there is often a range of actual ages at attendance. The number of growth measures per child may also be variable. Z-score-based methods often rely on analysis of the subset of individuals with complete data at all measurement occasions, thus reducing power and potentially introducing bias. Furthermore, if multiple growth measurements are included in regression models, these models can suffer from multicollinearity because of the strong correlations between measurements on the same individual.^11^ Finally, if associations between growth measures and a later outcome are conditioned on later growth measures, the coefficients are difficult to interpret and results can be affected by the reversal paradox.^12^

In this paper, we outline the application of linear spline multilevel models to length/height and weight trajectories in childhood, providing guidance on the process. We use examples from five birth cohort studies (see Tables 1 and 2 for information about each cohort) from different generations and different geographical regions with varying levels of economic development: (1) The Avon Longitudinal Study of Parents and Children (ALSPAC), a cohort of children born in the early 1990s in the South-West of England, (2) Born in Bradford (BiB), a cohort of children born between 2007 and 2010, in a deprived city in the North of England, (3) a cohort of children born in 2004 in Pelotas, a city in the South of Brazil, (4) Generation XXI, a cohort of children born in 2005/2006 in the North of Portugal and (5) the Promotion of Breastfeeding Intervention Trial (PROBIT), a national cohort born in the late 1990s in Belarus. Differences in data structure across the cohorts have implications for the application of linear spline multilevel models. We describe the two cohorts with the greatest difference in data structure (ALSPAC, which has very large inter-individual variation in the number and ages at measurement and measures from two sources with differing accuracy, and the 2004 Pelotas cohort, which has defined follow-up visits with very little inter-individual variability in age at attendance and all measurements are made by researchers) in detail in the main body of the text. The other three cohorts are presented in full in the online-only supplement, with key details presented in the main manuscript when relevant.

**Table 1.** Characteristics of birth cohorts.

ALSPAC	Born in Bradford (White British)	Born in Bradford (Pakistani)	Generation XXI	Pelotas 2004 cohort	PROBIT
Total sample size included in growth analysis	14,048	613	777	5282	4188	17,046
% male	51.5	51.6	50.1	50.5	51.8	51.8
% preterm birth (<37 weeks gestation)	6.1	6.1	4.6	7.2	14.0	–^a^
Mean maternal height in cm (SD)	164.0 (6.7)	164.0 (6.2)	159.4 (5.8)	160.5 (6.1)	158.7 (6.4)	164.4 (5.6)
% mothers who smoked in pregnancy	25.6	34.6	3.6	20.7	27.4	2.0

ALSPAC: The Avon Longitudinal Study of Parents and Children; PROBIT: the Promotion of Breastfeeding Intervention Trial.

a Eligibility criteria for PROBIT mean that all infants were term births (≥37 weeks gestation) with a birth weight ≥2.5 kg.

**Table 2.** Features of models for childhood growth in each birth cohort.

ALSPAC	Born in Bradford	Generation XXI	Pelotas 2004 cohort	PROBIT
Age range	0–10 years	0–2 years	0–6 years	0–4 years	0–5 years
Knot points for weight model	3 months, 1 and 7 years	4 and 9 months	10 days, 3 months, 1 and 3 years	3 months, 1 year	3 months, 1 year
Knot points for length/height model	3 months, 1 and 3 years	4 and 9 months	3 months, 1 and 3 years	3 months, 1 and 2 years	3 months, 1 year
Process for selecting knot points	(i) Identified fractional polynomial providing the best smooth curve, (ii) used this curve to identify approximate number and position of knot points, (iii) ran multiple models using alternative positions for knot points at ages in whole months, (iv) selected the combination of knot points giving the best model fit judged by log likelihood, (v) verified that model fit was not substantially compromised when choosing knot points at whole years	(i) Identified fractional polynomial providing the best smooth curve, (ii) used this curve to identify approximate number and position of knot points, (iii) ran multiple models using alternative positions for knot points at ages in whole months, (iv) selected the combination of knot points giving the best model fit judged by log likelihood	(i) Fit a model using the ALSPAC knot points, (ii) tested multiple models using alternative positions for knot points at ages in whole months around the ALSPAC knot points and verified that altering from the ALSPAC knot points did not improve model fit (judged by both log likelihood and differences between observed and predicted values), (iii) tested for improvement in model fit (judged by both log likelihood and differences between observed and predicted values) with the inclusion of an additional early knot point in the weight model	Knot points fit at planned follow-up ages	(i) Identified fractional polynomial providing the best smooth curve, (ii) fit knot points at planned clinic ages, (iii) chose the model which best fitted the fractional polynomial
Other covariates included in model^a^	Measurement source (measured vs. parent-reported)	Ethnicity and ethnicity interactions with splines, measurement source (research vs. health visitor)

ALSPAC: The Avon Longitudinal Study of Parents and Children; PROBIT: the Promotion of Breastfeeding Intervention Trial.

a All models included gender and interactions between gender and splines as fixed effects.

The paper is structured as follows. In Section 2, we describe multilevel models and their utility for modelling longitudinal data. In Section 3, we outline the cohorts used as illustrative examples in this paper, describing the country and setting in which they are based, and the growth data available. In Section 4, we outline the application of the growth models within our example cohorts, including a discussion of the process of model selection, any challenges we encountered in model convergence and model checking. Finally, in Section 5, we conclude with a discussion of the advantages and disadvantages of the linear spline multilevel approach compared with other growth modelling methods such as fractional polynomials, more complex spline functions and other non-linear models, as well as a consideration of the implications of our findings for the application of linear spline multilevel models in other cohorts with different data structures. We provide a suggested workflow for the implementation of these models, and sample Stata code.

## 2 Multilevel models for modelling growth trajectories

### 2.1 Multilevel models

Multilevel modelling is one approach that can be used to overcome some of the challenges in modelling longitudinal data. After selecting the appropriate function for age in order to model the average relationship between age and length/height or weight (or any other variable measured longitudinally^13^), individual-level (level 2) random effects for the intercept and age coefficient(s) capture each individual’s deviation from the average trajectory. These individual summaries of the growth trajectory can be extracted and related to later outcomes using a two-step process^14–17^ or in a single-step process in multivariate models.^13,18^ Individual-specific occasion-level (level 1) residuals are also estimated; these capture measurement error, that is, the deviation of observed measures from values predicted by the model.^19^

In contrast to z-score-based analysis approaches, multilevel modelling can capture the shape of growth over time, rather than relying on standardising the scale and variance of growth measures. The changing measurement error over time can be explicitly modelled. There is also more flexibility in terms of the data structure; there is no requirement for individuals to have been measured at the same ages,^19^ and varying numbers of measurements between individuals can be incorporated. All individuals with at least one observation can contribute to the model under the assumption that data are missing at random, that is, the probability of an observation being missing is related to other observed variables for that individual, but does not depend on the true value of the missing observation.^20^ A random slopes model for linear change in an outcome can be written as follows:

(1) yij=β0+u0j+β1×(age)ij+u1j×(age)ij+e0ij

where yij is the weight for individual *j* at time *i*, and

$e0\mathrm{ij}\;\sim N(0,\sigma_{e0}^{2})$ and (u0j,u1j) follow a bivariate normal distribution with means of zero and covariance

$$\Omega u=(\sigma_{u0}^{2}\sigma u01\sigma_{u1}^{2})$$

Here, β0 and β1(the ‘fixed’ coefficients) represent the average intercept and slope, respectively, and u0j and u1j (the ‘random’ coefficients) represent the deviation from the average intercept and slope, respectively, for individual *j*. The best linear unbiased predictions of these residuals are shrunk towards the mean, with estimates for individuals with greater variation (e.g. those with fewer growth measures) having greater shrinkage.^21^ The occasion-level residuals *e_0ij_* represent the measurement error, and have constant variance, but the model can be extended to incorporate a complex variance structure at the occasion level.^22^

### 2.2 Modelling non-linear growth

The simple multilevel model for growth shown above represents linear change in the outcome over time. For most biological processes, growth is non-linear. This non-linearity can be incorporated into multilevel models in several ways. One method would be to impose a transformation on either the growth measurements or on age, such that the relationship is approximately linear.^23^ This approach is, however, not very flexible and results in growth curves that are difficult to interpret. A more flexible approach is to model the non-linearity by including non-linear age functions in a multilevel model. This requires the best-fitting function of time to be selected. Quadratic or cubic models may fit the data well, if not, a broader range of curves could be considered by using fractional polynomial models; an approach that has been described in detail elsewhere.^24,25^ Briefly, a series of models are run using each of eight powers of age (−2, −1, −0.5, 0, 0.5, 1, 2, 3, where a power of zero is the log function), followed by models incorporating each combination of pairs of these powers. For more complex curves, all combinations of multiple powers can also be compared.^26^ The best fitting of these models is then selected, often by comparing the deviance across each model. The equation for a model with two powers of age, *p*_1_ and *p*_2_, in a multilevel framework is as follows:

(2) yij=β0+(β1+u1j)tijp1+(β2+u2j)tijp2+u0j+eij ifp1≠p2yij=β0+(β1+u1j)tijp1+(β2+u2j)tijp2logtij+u0j+eijifp1=p2

where *β*_0_, *β*_1_ and *β*_2_ are the fixed coefficients describing the average shape of the trajectory, and *u_kj_* describe the deviation of individual *j*’s trajectory from this average.

### 2.3 Linear spline multilevel models

A well-fitting multilevel model estimating a curve can be very useful for describing the average pattern of growth, or for assessing the relationship between early exposures and later growth. However, such models are not very conducive to exploring associations between growth and later outcomes, or for comparing growth across populations, since the polynomial terms (and their associations with other variables) are not easily interpreted. One approach that can yield more interpretable growth coefficients is to use a series of linear splines, joined at ‘knot points’, to model the growth trajectory. These models have also been referred to as piecewise linear or broken stick. As an example, a multilevel linear spline model for weight with knots at 3 and 12 months would allow different linear slopes from 0 to 3 months, 3 to 12 months and beyond 12 months, with these slopes varying between individuals.

We define *c* knot points at times *t_k_*, *k* = 1,…,*c*, and define *t*_0_ = 0, *t_c_*_ + 1_ = max(time). For person *j*, with weight*_ij_* observed at time *t_ij_* we create *c*+1 splines *s_ijk_*

For *k* = 1,…,*c*: *s_ijk_* = 0 if *t_ij_* ≤ *t_k_*_−1_

Sijk=tij-tk-1iftij≤tk

Sijk=tk-tk-1iftij>tk

In the multilevel context, a model with *c* knots would then be of the form

(3) $$y\mathrm{ij}=\beta 0+u0j+\sum_{\text{k}=1}^{\text{c}+1}(\beta \text{k}+u\mathrm{ki})s\mathrm{ijk}+e\mathrm{ji}$$

where *β*_0,…,_ *β_c_*_+1_ are the fixed coefficients describing the average intercept and average slope between each set of knots, *u_kj_* describe the deviation for individual *j* from the average slope between knots *k* − 1 and *k* and *u*_0_*_j_* is the deviation of individual *j*’s intercept from the average intercept.

Several methods for selecting the number and position of knot points are available. We have previously used fractional polynomials to derive a smooth function for the curve, and used the derivatives of this curve to decide the number and position of the knot points.^8,15^ Another possibility would be to start with a large number of knot points, gradually reducing the number until a ‘smooth’ curve is achieved. Other options could be to place knot points at the centiles of the distribution of age, or use stepwise regression to select knots where there is statistical evidence of a difference between the linear slopes either side of the knot point.^27^ Subject knowledge of the underlying biology of growth patterns may also decide the choice of knot point positioning, as may the availability of data – studies with few measurement occasions may only be able to place knot points at the mean age of each data collection. In Section 3, we describe the process of knot point selection in our example cohorts.

## 3 Description of example birth cohort studies

In this paper, we describe the application of linear spline multilevel models to child growth data in the ALSPAC and in the Pelotas 2004 cohort. Details of these two cohorts are provided below. Complete details of the remaining three cohorts from which we draw examples (BiB,^28^ Generation XXI and PROBIT^29^) are provided in the online supplement; brief details are shown in Tables 1 and 2, and are discussed throughout the paper where insights can be drawn that are not possible from ALSPAC and the Pelotas 2004 cohort.

### 3.1 The ALSPAC

#### 3.1.1 Description of cohort

The ALSPAC is a prospective cohort study investigating the health and development of children in the South-West of England, complete details of which are published elsewhere.^30,31^ Pregnant women residents in one of three Bristol-based health districts with an expected date of delivery between 1 April 1991 and 31 December 1992 were invited to take part in the study. Of these women, 14,541 were recruited. From these pregnancies, there were 14,062 live-born children, 13,988 of whom were alive one year. Follow-up has included parent- and child-completed questionnaires, links to routine data and clinic attendance.

#### 3.1.2 Description of child growth data

Length/height and weight data for the children are available from several sources. Birth length and weight are available for most children. Between birth and age 5 years, measures are available from routine child health clinics for most children, and from research clinic measurements on a random 10% subsample of the cohort. All cohort members were invited to research clinics from age 7 onwards. Across all ages parent-reported measures are available. Complete details of the measurement protocols are provided in the web supplement.

After the exclusion of multiple births (whose growth rates differ considerably from singletons), at least one growth measurement is available for 14,048 children. These children have a combined total of 106,933 length/height measurements and 120,081 weight measurements (median number of measurements per individual 7 for length/height and 8 for weight, interquartile ranges 5 to 9 and 5 to 11, respectively; minimum and maximum number of measurements per child were 1–36 for height and 1–34 for weight).

### 3.2 The Pelotas 2004 birth cohort

#### 3.2.1 Description of cohort

From 1 January 2004 to 31 December 2004 inclusive, a population-based birth cohort study attempted to enrol all births from mothers residing in the urban area of the city of Pelotas, Southern Brazil. Births were identified by daily visits to the five maternity hospitals. Mothers were interviewed soon after delivery. Of the 4263 live births in Pelotas during 2004, 4231 were enrolled in the cohort study. Follow-ups were done at home at mean (SD) ages 3.0 (0.1), 11.9 (0.2), 23.9 (0.4) and 49.5 (1.7) months; note the small inter-individual variation in the ages at follow-up occasions, which was made possible by the strategy of visiting children in their own homes and planning the visits individually to be as close to the target follow-up age as possible. Further information about the methodology of the 2004 Pelotas birth cohort study is described in detail elsewhere.^32^

#### 3.2.2 Description of child growth data

In addition to birth length and weight, length/height and weight were measured at each follow-up; see supplementary material for detailed protocols.

The sample size for inclusion in this paper is 4188 singletons, with a combined total of 19,721 length/height measurements and 19,688 weight measurements (median number of measurements per individual 5 for both length/height and weight, interquartile range 5 to 5 because most children attended all follow-ups).

### 3.3 Comparisons between the cohorts

In addition to differences in year of birth, socioeconomic factors, etc. between the cohorts (Table 1), there are data differences that have implications for the application of linear spline multilevel models. Within ALSPAC, growth measurements are available from routine health records, from research clinics and from parent-completed questionnaires. The likely differential accuracy of these measurement sources must be taken into account within the model. The Pelotas 2004 cohort has defined follow-up ages of birth, 3 months, 1, 2 and 4 years. There is very little inter-individual variation in the ages at which children were measured. This is in stark contrast to all of the other four cohorts, which have a much broader spread of ages at measurement. This has implications for model development – in Pelotas 2004 there is little option but to fit the knot points at or around the target ages for follow-ups, whereas in the other four cohorts, models can be tested and compared placing the knot points at various ages. PROBIT combines research clinic and routine healthcare measurements, with very high levels of participation. The BiB cohort participants are approximately 50% of white ethnicity and 50% of Pakistani ethnicity. Given the likely differences in growth trajectories between these groups, it is appropriate to model this from the outset. Generation XXI has the greatest frequency of measurements in all the five cohorts. The density of measurements in early infancy means that an additional knot point in the first weeks of life can be included.

## 4 Application of linear spline multilevel models for childhood growth

### 4.1 Selection of the number and position of knot points

**Figure 1.**
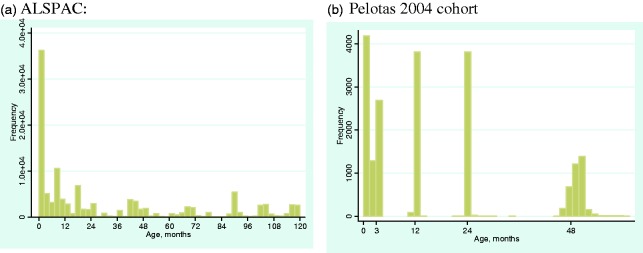
Distribution of the ages at measurement in (a) ALSPAC and (b) Pelotas 2004 cohorts. a. ALSPAC: b. Pelotas 2004 cohort ALSPAC: The Avon Longitudinal Study of Parents and Children. Note: Illustrating the greater inter-individual variability in ages at measurement in ALSPAC compared with the Pelotas 2004 cohort. Other cohorts are shown in Supplementary Figure 1.

The differing data structures necessitated a different strategy to identify and select the knot points within each cohort (Figure 1, Supplementary Figure 1, Table 2). Within ALSPAC, growth measurements were available over most of the age range birth to 10 years owing to the use of routine health visitor measurements and the wide variability in ages at clinic attendance and completion of questionnaires. Thus, there was very little restriction in where the knot points for the linear spline model could be placed. The approach used in this cohort was to estimate the curve that best described the growth data using fractional polynomials, and use this to estimate the approximate number and location of knot points. The best-fitting fractional polynomial was established for both length/height and weight by comparing the log likelihood values from models containing one or two age terms. For both height and weight, the best-fitting fractional polynomial had the age powers age^3^ and age^1/2^. For weight in males, the fixed part of the equation for this best-fitting fractional polynomial was

weightij=β0+β1(tij)3+β2(tij)1/2

where

*t_ij_* = age (weeks divided by 10) +0.01 at time *i* for individual *j*

*β*_0_ = 2.92 (SE = 0.023)

*β*_1_ = 0.0007 (SE = 0.0000004)

*β*_2_ = 3.02 (SE = 0.006)

Note that 0.01 is added to all ages in order to make all ages above zero because some of the fractional polynomial models include log(age) terms. The mean trajectory indicated by the best-fitting fractional polynomial models for length/height and weight in males for the ALSPAC cohort are shown in Supplementary Figure 2. These models indicated that for both weight and length/height, there appeared to be a phase of rapid growth in the first few months of life followed by a slightly slower rate of growth for the rest of infancy (up to approximately 1 year). For length/height, this was followed by a slightly slower rate of growth between about 1 and 3 years and a slower still rate of growth after about 3 years of age. For weight, the rate of growth seemed to increase after about age 7. In order to select the number and position of knot points, we fit linear spline models with all combinations of the following knot points:

Length/Height: early infancy knot point at 2, 3, 4, 5 or 6 months, late infancy knot point at 8, 9, 10, 11, 12, 13, 14, 15 or 16 months and early childhood knot point at every one month interval between 24 and 48 months.

Weight: early infancy knot point at 2, 3, 4, 5 or 6 months, late infancy knot point at 8, 9, 10, 11, 12, 13, 14, 15 or 16 months, early childhood knot point at every one month interval between 24 and 48 months and late childhood knot point at every one month interval between 60 and 108 months.

The ‘best-fitting’ model was selected on the basis of comparing the log likelihoods from each of these models, for males and females separately. This identified the following knot points: for weight for boys knots were birth, 4, 11 and 80 months and for girls were birth, 4, 10 and 80 months; for length/height for boys knot points were birth, 3, 10 and 29 months and for girls were birth, 2, 11 and 32 months. However, for ease of interpretation, presentation of results, and combination of males and females in analyses, we sought to simplify the model. Thus, we assessed whether the following knot points resulted in adequate model fit in a model combining males and females, but allowing different average intercepts and slopes by gender: knot points at 3 months, 1 year and 3 years for length/height, and 3 months, 1 year and 7 years for weight. This simplification of the model did not result in appreciably worse model fit (Supplementary Table 1).

Within the Pelotas 2004 cohort, growth measurements are only available from follow-ups, around which there is relatively little variability in the participants’ ages at attendance (Figure 1). We therefore fit models in the Pelotas data with the knot points at the planned ages of follow-ups (three months, one year and two years). The slopes for the rate of weight gain between 1 and 2 years and 2 and 4 years were almost identical. Therefore, the final model for weight was chosen as having two knot points, at three months and one year.

Knot points for BiB and Generation XXI were selected similar to ALSPAC. Knot points of 4 and 9 months were found to fit the data best in BiB. In Generation XXI, in addition to 3 and 12 months, a knot point in very early infancy (10 days) was included in the weight trajectory model in order to improve model fit. A slightly different process of knot point selection was used in the PROBIT, which aimed to select the knot points leading the best fit to the fractional polynomial model. Complete details for these three cohorts are provided in the supplementary material.

### 4.2 Including covariates

In all cohorts, interactions between gender and the intercept and each slope were fitted. Within BiB, interaction terms allowed different average intercepts and slope for White British and Pakistani ethnic groups. Within ALSPAC and BiB, measurements were available from multiple sources with different measurement error. In both cohorts, a dichotomous indicator of measurement source was included in the models as a fixed effect, representing measured (research clinic or health worker) versus parent-reported in ALSPAC, and research clinic versus health worker in BiB. Separating the research clinic and health worker measurements in the ALSPAC model did not improve model fit and so was deemed unnecessary.

The growth trajectory in the ALSPAC cohort, with separate intercepts and slopes for males and females and a fixed effect for measurement source, can therefore be represented by the following equation:

(4) $$y\mathrm{ij}=\beta 0+\beta 1(\text{male})+u0j+\sum_{k=1}^{c+1}(\beta k+\beta l(\text{male})+u\mathrm{ki})s\mathrm{ijk}+\beta 3(\text{source})\mathrm{ij}+e\mathrm{ji}$$

where *β*_0_ is the fixed coefficient describing the average intercept in females, *β*_1_ is the fixed coefficient describing the difference in average intercept between males and females, *β_k_* are the fixed coefficients describing the average linear slopes in females, *β_l_* are the fixed coefficients describing the difference in average linear slopes between males and females, *β*_3_ is the fixed coefficient describing the average difference for measurements from parent-reported questionnaires compared with those from research clinics or health workers, *u_kj_* describe the deviation for individual *j* from the average slope between knots *k* − 1 and *k*, and *u*_0_*_j_* is the deviation of individual *j*’s intercept from the average intercept.

### 4.2 Complex level 1 variation

Since the scale and measurement error of height and weight vary over time, it is necessary to measure the potentially complex nature of level 1 variation. We did this by allowing the within-individual (occasion level, level 1) variances to vary with time. The way in which complex level 1 variation is modelled varies between outcomes and datasets, and can make a considerable difference to model convergence and model fit. Possible approaches to parametrising age as a level 1 random effect include as a continuous term, or as each of the linear splines. The details of which functions of age were modelled as complex level 1 variation in each model are provided in the supplementary material.

Inclusion of an occasion-level random effect for the measurement source variable in ALSPAC or BiB, gender in all cohorts, or ethnicity in BiB did not improve the differences between observed and predicted measurements, so fixed effects were decided to be sufficient. In each model, we assumed that there was no correlation between the occasion-level random effects. Thus the occasion-level variance/covariance matrix had all off-diagonal terms set to zero.

### 4.3 Modelling the variance-covariance matrix of individual-level random effects

We put no constraints on the individual-level variance/covariance matrix for most models. However, for both the Pelotas and BiB length/height models, we could not estimate the covariance between the individual-level random effects for birth length and growth between 0 and 3/4 months. If these covariances were estimated, the model estimated a negative variance for the level 2 random effects for growth between birth and 3/4 months. Similarly, the covariance between birth weight and weight gain between 0 and 10 days could not be estimated in Generation XXI. This possibly reflects the lower frequency of measurement occasions in Pelotas, and the lack of measured birth length in BiB. Variation between individuals increased gradually over time, whereas that within individuals increased sharply from birth and more slowly after 1 year.

### 4.4 Model checking

For all cohorts, most individuals had at least one growth measurement within each time period defined by the linear splines (Supplementary Table 2). One exception was the length trajectories for BiB, where birth length was not measured. For the purposes of comparison in this paper, we have estimated the length trajectories from birth for BiB, but it should be noted that the estimates for birth length are less reliable in BiB than in the other cohorts due to the lack of measured birth length. In order to check the influence of missing birth length on this model, we also fitted models with age centred at 2 weeks and at 1 month; model fit was similar and estimated mean growth rates in the first period were also similar.

Model fit, as judged by the differences between observed growth measurements and those predicted by the models, was good in all models (Table 3 and Supplementary Table 3). For all cohorts, the individual- and occasion-level residuals were approximately normally distributed (Figure 1 and Supplementary Figure 3). There is some evidence that deviation from normality increased as measurements became more sparse, generally as the children get older within each cohort.

**Table 3.** Differences between observed measurements and those predicted by the multilevel model for ALSPAC.

Mean actual measurement (SD)	Mean predicted measurement (SD)	Mean difference (SD)
**ALSPAC, *N* = 13,922**
Length/height models
Birth length	50.61 (2.36)	50.72 (1.65)	−0.11 (1.19)
>0 to ≤3 months	57.25 (3.11)	57.18 (2.66)	0.07 (1.33)
>3 months to ≤1 year	69.95 (4.78)	70.06 (4.39)	−0.11 (1.38)
>1 year to ≤3 years	83.64 (5.56)	83.61 (5.12)	0.02 (1.48)
>3 years to ≤10 years	117.70 (15.26)	117.65 (15.00)	0.05 (1.71)
Weight models
Birth weight	3.40 (0.55)	3.36 (0.47)	0.04 (0.15)
>0 to ≤3 months	4.61 (0.91)	4.65 (0.87)	−0.04 (0.17)
>3 months to ≤1 year	8.49 (1.48)	8.45 (1.44)	0.05 (0.27)
>1 year to ≤7 years	15.28 (4.26)	15.29 (4.11)	−0.01 (0.79)
>7 years to ≤10 years	29.41 (6.48)	29.42 (6.14)	−0.01 (1.06)

ALSPAC: The Avon Longitudinal Study of Parents and Children.

We plotted the residuals from a linear regression between consecutive level 1 residuals for the same individual against the time difference between the consecutive measures (Supplementary Figure 4) and saw no evidence of autocorrelation. In order to verify that models were not dominated by individuals with either very few or very many growth measures (those with many measures in particular may have different growth patterns compared with the average, e.g. they may be measured frequently because of failure to thrive), sensitivity analysis were conducted excluding individuals with less than the 25th centile number of measurements or more than the 75th centile number of measurements. As an example, within the ALSPAC models this resulted in coefficients and individual-level residuals that had correlations ≥0.90 compared with the model including all individuals. An alternative check that we could have performed to check this assumption would be to only include a certain number of measurements for individuals with many measures.

### 4.5 Growth patterns within each birth cohort study

Within each cohort, similar patterns of growth were seen such that rapid length/height growth and weight gain in early infancy were followed by a slowing down of growth in later infancy and early childhood (Table 4 and Supplementary Table 4). In ALSPAC, where data are available for older ages than the other cohorts, rates of weight gain began to increase again between ages 7 and 10 years (Supplementary Figure 4). Rates of growth are broadly similar across the cohorts; the greatest differences are seen at birth, with greater similarity between the cohorts in terms of postnatal growth.

**Table 4.** Growth rates predicted by linear spline multilevel models for girls.

Mean (SD) growth rates
ALSPAC	Born in Bradford (White British ethnicity)	Born in Bradford (Pakistani ethnicity)	Generation XXI	Pelotas 2004	PROBIT
**Length/Height**
Girls*, N*	6731	316	396	2465	2018	7905
Birth length (cm)	50.00 (1.57)	48.36 (1.16)	47.91 (1.04)	48.30 (1.86)	47.77 (1.87)	51.42 (1.66)
Early infancy (cm/month)	3.57 (0.16)	3.54 (0.39)	3.92 (0.36)	3.79 (0.22)	3.83 (0.31)	2.96 (0.45)
Late infancy (cm/month)	1.64 (0.15)	1.63 (0.27)	1.59 (0.25)	1.70 (0.17)	1.59 (0.15)	1.77 (0.17)
Early childhood (cm/month)	0.83 (0.07)	0.94 (0.05)	0.95 (0.05)	0.90 (0.08)	1.04 (0.12)	0.76 (0.04)
Later childhood (cm/month)	0.53 (0.04)	N/A	N/A	0.53 (0.02)	0.65 (0.05)	N/A
**Weight**
Girls*, N*	6731	316	396	2616	2018	7905
Birth weight (kg)	3.30 (0.44)	3.16 (0.51)	2.98 (0.47)	3.11 (0.40)	3.10 (0.33)	3.33 (0.31)
Early neonatal (kg /month)	N/A	N/A	N/A	0.42 (0.64)	N/A	N/A
Early infancy (kg /month)	0.88 (0.14)	0.83 (0.17)	0.82 (0.15)	0.87 (0.16)	0.81 (0.11)	0.87 (0.11)
Late infancy (kg /month)	0.45 (0.09)	0.44 (0.12)	0.42 (0.13)	0.40 (0.09)	0.37 (0.08)	0.53 (0.07)
Early childhood (kg /month)	0.19 (0.04)	0.22 (0.04)	0.24 (0.05)	0.22 (0.05)	0.23 (0.05)	0.15 (0.02)
Later childhood (kg /month)	0.32 (0.10)	N/A	N/A	0.19 (0.05)	N/A	N/A

ALSPAC: The Avon Longitudinal Study of Parents and Children; PROBIT: the Promotion of Breastfeeding Intervention Trial; BiB: Born in Bradford.

Growth rates for early neonatal, early infancy, late infancy, early childhood and late childhood, respectively: ALSPAC length/height: n/a, 0–3 months, 3–12 months, 1–3 years, 3–10 years; ALSPAC weight: n/a, 0–3 months, 3–12 months, 1–7 years, 7–10 years; BiB length and weight: n/a, 0–4 months, 4–9 months, 9–24 months, n/a; Generation XXI length/height: n/a, 0–3 months, 3–12 months, 1–3 years, 3–6 years; Generation XXI weight: 0–10 days, 10 days–3 months, 3–12 months, 1–3 years, 3–6 years; Pelotas length/height: n/a, 0–3 months, 3–12 months, 1–2 years, 2–4 years; Pelotas weight: n/a, 0–3 months, 3–24 months, 2–4 years, n/a; PROBIT length/height and weight: n/a, 0–3 months, 3–12 months, 1–3 years, 3–5 years.

The variances and covariances of the level 2 random effects show broadly similar patterns across all cohorts (Supplementary Table 5).

## 5 Discussion and relevance for other studies

### 5.1 Application of linear spline multilevel models for childhood growth

#### 5.1.1 Model fit and similarity of knot points across cohorts

Within each cohort, linear spline multilevel models provided good fit to the observed data. Similar knot points summarised the growth trajectories in all five cohorts; faster growth was observed in the first few months of life, followed by slower growth for the rest of the first year of life and an even slower growth rate later in childhood. The similarity of the knot points selected in each of these diverse cohorts provides some external validity to the model and reassurance that these models are identifying periods of growth which are biologically, as well as statistically, distinct. The knot points identified in these five cohorts could be used as a starting point for model selection in the application of this methodology to other datasets. The analyses we present here also provides reassurance that routinely collected growth measurements can be successfully used to model child growth in epidemiological studies, or to supplement research measurements of growth in order to minimise data collection costs.

The frequency and intensity of data collection within each cohort had implications for the application of the linear spline multilevel models. Where data had been collected at a wide range of ages across the cohort members, alternative knot point positions could be tested to select the best-fitting model. A variety of methods could be used to select the knot points (see Box 1: suggested workflow), but care should be taken in this process, for example, knot points should not be placed too close together in such a way that few individuals have measurements between the knot points. In cohorts with fewer measurement occasions, knot points had to be fixed at planned follow-up ages. However, the analyses of the ALSPAC cohort demonstrated that the exact position of the knot points does not always have a strong effect on model fit, suggesting that knot points could be varied depending on data availability and/or the research question of interest. Given that the exact position of the knot points had only limited consequence, we would advise not to interpret the exact growth periods with too much certainty – rather, we would suggest that they should be interpreted as approximate periods of the life course, for example, early infancy, late infancy, early childhood, mid childhood, etc. In the Generation XXI cohort, where most children had several measures of growth taken in the first weeks of life, good model fit could only be achieved if an additional knot point was included at age 10 days, modelling slow weight gain or neonatal weight loss in early life. These early weight changes could not be modelled in the other cohorts with fewer early life measurements; in the other four cohorts, a constant rate of weight gain had to be assumed between birth and 3/4 months of age.

**Box 1.** Suggested workflow for the application of linear spline multilevel models.

1. Choose approximate knot point locations. Possible strategies include the following:
a. Use knowledge of the underlying biology of growth patterns to decide the choice of knot point positioning.
b. Identify the best-fitting curve (using fractional polynomials or other method) and use the derivatives of the curve to identify the number and approximate timings of knot points.
c. Identify the best-fitting curve (using fractional polynomials or other method) and visually estimate the number and approximate timings of knot points.
d. Start with a large number of knot points, gradually reducing the number until a ‘smooth’ curve is achieved.
e. Place knot points at the centiles of the distribution of age.
f. Use stepwise regression to select knots where there is statistical evidence of a difference between the linear slopes either side of the knot point.
g. For datasets with a small number of follow-up occasions and limited inter-individual variability in the timing of follow-ups, knot points can be placed at the target ages of follow-up occasions.
h. When modelling child growth data, it may be possible to start with the knot points identified in the five cohorts shown in this paper (i.e. 10 days in data permits, then 3–4 months, 9–12 months, 3 years (height) and 7 years (weight)).
2. Test various models to determine the final knot point locations. We suggest that this choice should be a combination of model fit (which may be assessed by likelihood values, other model fit statistics, residual standard deviation, differences between observed and predicted measurements, etc.) and interpretability.
3. Identify which covariates will be included and how these will be modelled, for example, as fixed effects only or as random effects, with or without interactions between the covariates and the linear splines.
4. Determine whether it is necessary to model complex level 1 variation (e.g. this will be more important when the variance of the outcome changes over time) and how best to model this (e.g. age could be modelled as a continuous term or as each of the linear splines) – comparisons of model fit will be important in this process.
5. Perform model checks. Potential model checks include the following:
a. Examine the difference between observed and predicted measurements (range, mean and reference range) overall and in different periods of time to check whether model fit is consistent across the time range of the model.
b. Assess the normality of level 1 and level 2 residuals.
c. Plot the difference between consecutive level 1 residuals for the same individual against the time difference between the consecutive measures as a check for autocorrelation. If autocorrelation is detected, some statistical software packages can model this.
d. Depending on the nature of the dataset, it may be prudent to conduct various sensitivity analyses, for example, verify that models are not dominated by individuals with a greater than average number of measurements, verify that the selected knot points are appropriate for all sub-groups of the population, verify that model fit is similar for all sub-groups of the population, etc.

#### 5.1.2 Modelling complex level 1 variation

Level 1 variation can arise not only from measurement errors due to instrument or recording errors, but also due to day-to-day fluctuations. Both of these types of measurement error vary with age – it is far more difficult to measure the length of a young baby than to measure standing height in an older child, and day-to-day fluctuations in weight are likely to represent a greater proportion of a baby’s total weight compared with a young child. Knowing about the likely structure of these measurement errors can inform the choice of modelling for the level 1 variation. However, comparison of model fit between alternative specifications is likely to be important when determining the optimal level 1 variance structure – for example, there was variability between our models in whether including a linear age term or all linear splines in the level 1 variance provided the best combination of convergence, model fit and plausible (positive) estimates of all level 2 random effect variances. There is some evidence that misspecification of complex level 1 variation has little influence on the fixed effects in a multilevel model,^33^ but it can affect the level 2 residuals.

#### 5.1.3 Software

In our examples, all analyses were conducted using the runmlwin command^34^ in Stata version 12,^35^ which calls the MLwiN program.^36^ Other software packages, for example, Stata alone (without calling on MLwiN), SAS and R are able to perform similar modelling, but may not have some of the flexibility offered by MLwiN. For example, within Stata and R it is not possible to allow complex level 1 variation. We provide example Stata syntax for these models in the online supplementary material.

#### 5.1.4 Further developments

In our analyses, we observed some non-normality in the level 1 residuals. Although there is some evidence that this has limited impact on the fixed effects of such models,^37,38^ one possible further development could be to model different distributions for the level 1 residuals. The methods used here allow for correlation between growth measures at different times to depend on the individuals’ underlying birth size and growth trajectory. They do not allow for autocorrelation beyond this – where for example, measures may have a greater correlation than that predicted by the model because of a period of illness or a short-term growth spurt. Autocorrelation can be modelled using standard software when the intervals between repeated measures are regular; but this is more complex when there are irregular gaps between measurement occasions: it is then necessary to parametrise the autocorrelation function explicitly.^39^

### 5.2 Linear spline multilevel models and other models for childhood growth

Linear spline multilevel models assume biologically implausible piecewise linear growth. However, this permits a simplification of the growth trajectories. Using these models, linear growth rates in different periods of childhood can be compared across populations, which is simpler to present and understand than describing differences in more complex non-linear growth functions. The linear spline multilevel modelling approach is particularly useful for exploring the associations between childhood growth and later outcomes. Regression models for the association between linear growth rates in different periods of childhood and later outcomes are more easily interpretable^14–17^ than coefficients for polynomial age terms.

Other methods are available for modelling childhood growth, which do not assume the biologically implausible piecewise linear structure. Apart from the fractional polynomial models mentioned in Section 2, other approaches to modelling a curved function for growth include (i) non-linear splines,^40^ (ii) superimposition by translation and rotation, in which individual growth curves are brought towards the mean curve by three parameters that shift the curve up or down, left or right, and stretch or squash the age axis^41^ and (iii) Preece–Baines models, which involve fitting many curves and result in five parameters, thus having relatively few degrees of freedom.^42^ From the non-linear splines models or fractional polynomial curves, it is possible to extract features of the individual curves, for example, the age and magnitude of peak height velocity, or if body mass index (BMI) was the growth process being modelled, features of interest might include the BMI peak in infancy or the adiposity rebound (BMI nadir that occurs at approximately age six years). The summary measures of interest must be defined before commencing analysis.

Thus, compared with curvilinear models for growth, the main advantage of the linear spline approach is its interpretability, whilst its main disadvantage is the non-plausibility of the piecewise linear shape. Compared with simpler z-score-based approaches to modelling growth, some of the key advantages are that multilevel modelling allows the shape of the trajectory to be modelled, does not require complete and balanced data for all individuals and correctly models the non-independence between repeat measures on the same individual. Multilevel modelling does, of course, have some disadvantages compared with simpler z-score approaches. It requires specialist software and statistical knowledge, whereas z-score analysis can be done by many analysts on standard software. The balance of advantages and disadvantages of multilevel modelling compared with simpler methods will depend on several factors. Firstly, for cohorts utilising growth data collected as part of routine health care, or where ages at attendance at planned research clinic follow-ups are very variable between individuals, z-score-based methods will be more difficult to apply, and multilevel models will considerably increase power and efficiency. Likewise, similar benefits to using multilevel models will apply if there is a lot of missing data. The larger the number of measures per individual, and the closer together the measurements are over age, the greater the extent multilevel models will reduce the dimensionality of the growth data and avoid multicollinearity compared with z-score approaches.

### 5.3 Between cohort differences in growth patterns

The World Health Organization (WHO) developed a ‘growth standard’ for child growth, by comparing growth patterns of children from a total of 8500 children from six countries between 1997 and 2003 (Brazil, Ghana, India, Norway, Oman and the United States). In order to be included in the study, infants needed to be delivered at term, breastfed, suffering from no illnesses, from high socioeconomic families and have mothers who did not smoke. In this healthy, affluent population from diverse countries, the WHO described very similar growth patterns in children from all the countries involved in their study.^6^ In our analyses, despite the considerable demographic, social and economic differences across these five cohorts, and despite the almost 20 years gap in the birth years of the two UK-based cohorts, patterns of growth are remarkably similar across the populations. The growth rates we observe are also similar to those seen in the WHO study. For example, the WHO reported a median rate of growth between birth and three months of 0.996 kg/month for males and 0.868 kg/month for females. This compares with the mean rates of growth in this period observed in each of our cohorts (e.g. 1.04 kg/month in ALSPAC males, 0.87 kg/month in Generation XXI females). With the exception of PROBIT, which excludes preterm and low birth weight infants, these cohorts are general population studies. Unlike in the WHO study, there is a range of levels of disadvantage within each cohort, and many of the children included in our analysis were not breastfed, not term deliveries and suffered from childhood illnesses. ALSPAC participants tend to be socioeconomically advantaged compared with the source population, and hence growth rates in the ALSPAC cohort may not necessarily generalise to the whole population of Avon. However, the Pelotas cohort had extremely high participation rates and does include some very low socioeconomic status individuals. Thus it is reassuring that even in the presence of these complexities, similar average growth rates are seen across the different cohorts. The most marked differences between the cohorts included in our analyses were present at birth. Whilst genetic, maternal nutrition, maternal smoking and other intrauterine factors may play a part, these differences are perhaps most likely to be attributable to differences in maternal size; maternal height has been shown to explain the majority of the maternal education differences in length/height growth within both ALSPAC^43^ and the 2004 Pelotas cohort,^9^ as well as the ethnic differences in birth size (but not postnatal growth) in BiB.^44^

### 5.4 Conclusions

Linear spline multilevel models provide a useful method to summarise growth trajectories, reducing the dimensionality of the data in cohorts rich in growth measurements, and providing interpretable growth summaries that can be used to compare growth rates across populations, assess associations between early life exposures and child growth or examine associations between child growth and later outcomes. In five cohorts from different geographic regions and birth years, a good level of model fit was achieved with similar knot points. This provides reassurance that the models are identifying periods of growth with biological relevance, and provides a suggested starting point for model estimation in other cohorts. A suggested workflow for the implementation of linear spline multilevel models is shown in Box 1.

**Figure 2.**
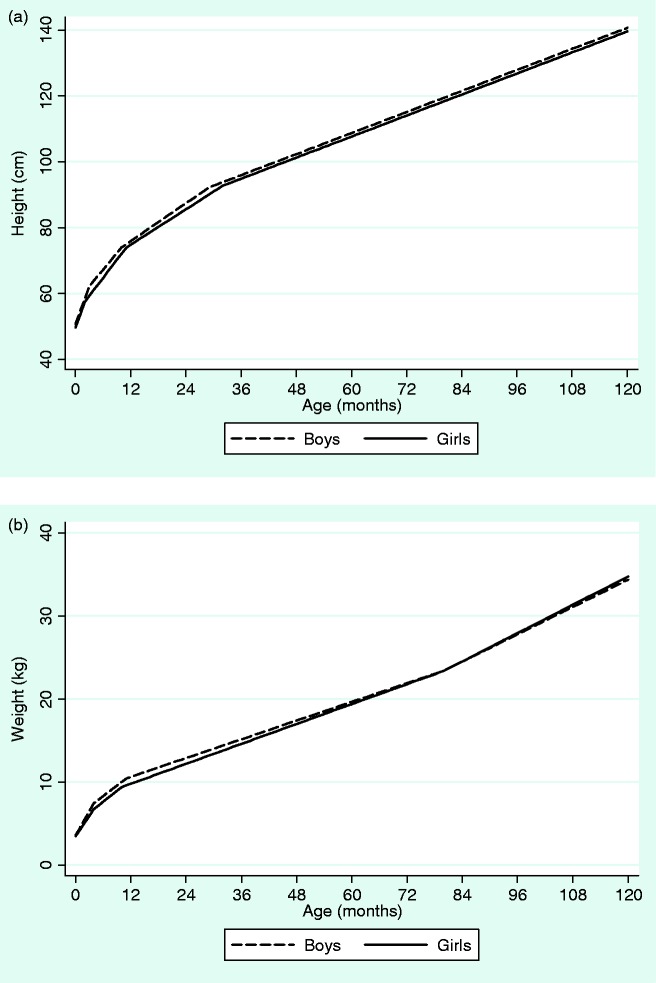
Average predicted length/height and weight trajectories from the ALSPAC cohort; (a) Mean predicted height trajectories for males (dashed line) and females (solid line) in ALSPAC and (b) Mean predicted weight trajectories for males (dashed line) and females (solid line) in ALSPAC. ALSPAC: The Avon Longitudinal Study of Parents and Children.

## Supplementary Material

Supplementary material
