# Supplementary material for: Linear spline multilevel models for summarising childhood growth trajectories: A guide to their application using examples from five birth cohorts
Source: Stat Methods Med Res. 2013 Oct 9;25(5):1854–74. doi: 10.1177/0962280213503925 (PMC4074455; doi:10.1177/0962280213503925)
Supplement: Supplementary material [file Supplementary_material_final.pdf]

Online only supplementary material for:

Linear spline multilevel models for the measurement of childhood growth: A guide to their application using examples from five birth cohorts

Laura D Howe[1], Kate Tilling[2], Alicia Matijasevich[3], Emily S Petherick[4], Ana Cristina Santos[5], Lesley Fairley[4], John Wright[4], Iná S. Santos[3], Aluísio J.D. Barros[3], Richard M Martin[2,6], Michael Kramer[7], Natalia Bogdanovich[8], Lidia Matush[8], Henrique Barros[5], Debbie A Lawlor[1]

1. MRC Integrative Epidemiology Unit at the University of Bristol, School of Social and Community Medicine, University of Bristol, UK
2. School of Social and Community Medicine, University of Bristol, UK
3. Postgraduate Programme in Epidemiology, Federal University of Pelotas, Pelotas, Brazil
4. Bradford Teaching Hospitals NHS Foundation Trust, Bradford, UK
5. Department Clinical Epidemiology, Predictive Medicine and Public Health, University of Porto Medical School, University of Porto Institute of Public Health, Porto, Portugal
6. University of Bristol / University Hospitals Bristol NHS Foundation Trust National Institute for Health Research Bristol Nutrition Biomedical Research Unit, Bristol, UK
7. Department of Pediatrics, Faculty of Medicine, McGill University, Montreal, Canada
8. Belarussian Ministry of Health and Belarussian Maternal and Child Health Research Institute, Belarus

Details of Born in Bradford, Generation XXI and Probit:

Born in Bradford (BiB)

Description of cohort

Born in Bradford (BiB) is a longitudinal multi-ethnic birth cohort study aiming to examine the impact of environmental, psychological and genetic factors on maternal and child health and wellbeing (1). Women were recruited while waiting for their oral glucose tolerance test, a routine procedure offered to all pregnant women registered at the Bradford Royal Infirmary at 26-28 weeks gestation. The full BiB cohort recruited 13,776 participants between 2007 and 2010 and the cohort is broadly characteristic of the city's maternal population.

A subsample of the BiB cohort (BiB 1000) recruited between August 2008 and March 2009 were invited to participate in a sub-study where infants were followed up with a visit at around 6, 12, 18 and 24 months of age. 1,917 women were eligible to be in this sub-study and 1,735 consented. Of these women, 1,707 had a singleton birth. These models will be extended to the full BiB cohort once data are available.

Description of child growth data

Weight and length measurements between birth and 2 years were available from two sources. The infants were visited at home or in clinics when the babies were around 6, 12, 18, and 24 months old. At these visits infants were weighed and length measured. Measurements were also available from health visitor records, which form part of standard care in the UK, for the majority of the cohort (98.8%, with mean ages at measurement of 6.7 months, 12.7 months, 18.7 months, and 25.3 months). The sample size for inclusion in this paper is 1,390 individuals with at least one length and weight measure, with a combined total of 5,472 length measurements and 11, 411 weight measurements (median number of measurements per individual 4 for length, 7 for weight, interquartile ranges 3 to5 and 6 to 9 respectively). Approximately half of the BiB cohort members are of white ethnicity; the other half are predominantly of Pakistani origin. Given this population stratification, ethnic differences in growth were modelled in BiB.

Generation XXI

Description of cohort

Geração XXI (Generation XXI) is a birth cohort study based in the city of Porto, Portugal. Recruitment took place between April 2005 and August 2006 at the five public maternity units (all level-three), covering 6 municipalities of the metropolitan area of Porto. Of the mothers invited to participate, 91.4% agreed. The total number of infants (gestational age ≥24 weeks) included in the cohort is 8,647. Four years after birth, the whole cohort was invited for a re-evaluation.

Description of child growth data

At the assessment four years after birth, mothers were asked to bring their child's health book for digitalization. This book is the mother's record of her child's contact with the routine health care services – she is asked to bring the book when taking the child to health centres, and information is recorded within it by health professionals. From the child health books, all data regarding measures of the child's weight and length/height between birth and 70 months of age were abstracted. These measurements were performed by health professionals as part of the standard child care in Portugal. Of the 7,458 participants who attended the follow-up evaluation, 2,076 children did not bring their child health book to the cohort assessment, excluding them from this data analysis. We have also excluded 100 twins from this study. Thus the sample eligible for analysis here is 5,282 children, with a combined total of 101,723 measurements of weight and 86,936 of length (median

number of measurements per individual 9 for length/height and 10 for weight, interquartile ranges 5 to 14 and 5 to 16 respectively).

Promotion of Breastfeeding Intervention Trial (PROBIT)

Description of cohort

The Promotion of Breastfeeding Intervention Trial (PROBIT) was a cluster-randomised trial in the Republic of Belarus (2), in which 31 maternity hospitals and each of their affiliated polyclinics (out-patient clinics for routine health-care follow-up) were randomly assigned to a breastfeeding promotion intervention or to usual practice. Overall, 17,046 full-term (≥37 weeks gestation), healthy singletons who weighed at least 2500 g, with Apgar score of at least 5 at 5 min, and who initiated breastfeeding, were recruited between June 1996 and December 1997. These children were followed up at polyclinic visits at 1, 2, 3, 6, 9 and 12 months; home visits were made when polyclinic visits were missed.

Description of child growth data

Birthweight was recorded during the postpartum stay. Weight and length/height were measured by paediatricians at the scheduled study visits (at 1, 2, 3, 6, 9 and 12 months) with over 93% of the children attending all six visits. After 12 months, children were seen approximately annually as part of routine clinical practice. The timing of these routine clinic assessments varied considerably between children and not all children attended all clinics. Measurements from the routine assessments were abstracted retrospectively from medical records by the child's paediatrician at around 6.5 years of age. The sample size for inclusion in this paper is 16,368, with a combined total of 139,427 length/height measurements and 140,207 weight measurements (median number of measurements per individual 9, interquartile range 7 to 10 for length/height, and 7 to 11 for weight).

Details of measurement protocols in ALSPAC and the Pelotas 2004 cohort:

ALSPAC:

Birth length (crown-heel) was measured by ALSPAC staff who visited newborns soon after birth (median 1 day, range 1-14 days), using a Harpenden Neonatometer (Holtain Ltd). Birth weight was extracted from medical records. From birth to five years, length and weight measurements were extracted from health visitor records, which form part of standard child care in the UK. In this cohort we had up to four measurements taken on average at six weeks, 10, 21, and 48 months of age, which previous work has shown to have good accuracy (3). For a random 10% of the cohort, we also have length/height and weight measurements from research clinics, held between the ages of four months and five years of age. From age seven years upwards, all children were invited to annual clinics. At the clinics between four months and five years, crown-heel length for children aged 4 to 25 months was measured using a Harpenden Neonatometer and from 25 months onwards standing height was measured using a Leicester Height Measure; weight was measured using Fereday 100kg combined scale (4 month clinic), Soenhle scale or Seca scale model 724 (8 month clinic), Seca 724 or Seca 835 (12 month clinic), Seca 835 (18 months onwards). From age seven years upwards, all children were invited to annual clinics, at which standing height was measured to the last complete mm using the Harpenden Stadiometer and weight was measured to the nearest 0.1kg using the Tanita Body Fat Analyser (Model TBF 305). Across all ages, parent-reported child length/heights and weights are also available from questionnaires.

Pelotas 2004:

Birth length was measured within 24 hours of delivery by trained research fieldworkers following standardized procedure using ARTHAG infantometers with 1mm precision (AHRTAG baby length measures, London). At each follow-up, anthropometric measurements were performed by trained fieldworkers with the children dressed in underwear and barefoot. Recumbent length (children ≤24 months of age) and standing height (48 months of age) were measured using a portable infantometer with 1mm precision, custom built for these studies.

Details of ethical approval for each cohort:

ALSPAC: Ethical approval for the study was obtained from the ALSPAC Law and Ethics Committee and the Local Research Ethics Committees.

BiB: Ethical approval for the data collection was granted by Bradford Research Ethics Committee (Ref 07/H1302/112).

Generation XXI: Generation XXI was approved by the Ethics Committee from Hospital Sao Joao and the University of Porto Medical School and by the Portuguese National Committee on Individual Data Protection.

Pelotas 2004 cohort: The perinatal study and each follow-up were approved by the Research Ethics Committee of the Federal University of Pelotas School of Medicine.

PROBIT: The institutional review board of the Montreal Children's Hospital approved the PROBIT trial, and participating mothers signed consent forms (in Russian).

Details of knot point selection in Born in Bradford, Generation XXI and Probit:

The same process as in ALSPAC was employed for selection of knot points in BiB, i.e. selection of the best-fitting fractional polynomial, using this to identify the approximate number and position of knot points, and then testing and comparing models with knot points placed at and around the expected ages. However, knot points at four and nine months fitted the data considerably better in BiB than the ALSPAC knot points of three and 12 months.

Within the Generation XXI cohort, we commenced the analysis by fitting a model using the ALSPAC knot points (3 months, 1 year and 3 years). Model fit from this model was then compared to using knot points at one month intervals either side of the ALSPAC knot point ages. Whilst the length/height model with knot points at three months, one year and three years fit the observed data very well, poor model fit was seen for the weight model, particularly for birth weight. Generation XXI has a far greater density of data than any of the other cohorts included in this analysis. Many of the infants had multiple growth measurements in the first month of life. One option we considered to improve model fit was to randomly select one measurement per child within any one month period (an approach which has successfully been applied to multilevel linear spline modelling of gestational weight gain (4)). This approach did not improve the fit of the predicted data to the observed measurements. However, when an earlier knot point was included in the model, birth weight and subsequent weights were all predicted well by the model. Models with knot points at ten days, two weeks and one month all converged, and the model with a knot point at ten days gave the best model fit. Models with a knot point earlier than ten days did not converge.

In PROBIT, fractional polynomial models were again used to identify the best fitting curve to describe the growth trajectories. Knot points for the linear spline models were then set at each planned follow-up age. Combinations of these knot points were assessed, such that the combination providing the best fit to the fractional polynomial was selected. The best-fitting model based only on the model deviance had knots at 6 and 12 months. However, we had a priori specified that we wanted the periods 0-1, 1-2, 2-3, 3-6, 6-12 and 12-60 months to have equal weighting in the knot-point selection. The best-fitting model with deviance in these periods equally weighted had knots at 3 and 12 months.

Modelling complex level 1 variation:

In most cohorts, a linear age term was included as an occasion-level random effect in order to allow the level 1 variance to change with time. In the weight model for PROBIT and the height model for ALSPAC, each of the linear splines was included in the level 1 variance instead of a linear age term. In both of these models, model fit was similar and level 2 residuals were very strongly correlated comparing models where variance terms for either linear age or all splines were estimated, but modelling the splines in the level 1 variance reduced the correlations between the individual-level residuals, which is helpful if the residuals are to be used as exposures for later outcomes. In other cohorts, some models would not converge when all linear splines were included in the level 1 variance, and some converged but resulted in estimates of some level 2 random effect variances as negative. For the Pelotas height model, including either all linear splines or a single linear age term in the level 1 variance resulted in the model estimating a negative variance for the level 2 random effect for growth between birth and three months. This was resolved by including a dichotomous indicator of age, estimating one variance for the level 1 random effects at birth and the three and 12 month follow-ups, and a different variance for follow-ups at 24 and 48 months.

Sample Stata syntax for estimating linear spline multilevel models using the ‘runmlwin’ command:

Notes:

This syntax utilises the user-written command ‘runmlwin’ which must be installed prior to use. The most recent version of MLwiN must be installed in order to be able to use this command. Alternatives to MLwiN include the xtmixed command in Stata, the procmixed command in SAS or various packages in R (e.g. lme). Explanatory notes (indicated by *) are provided above each line of syntax.

Data are in long format. Gender is included in this model as a covariate. No other covariates are included.

```
*Identify the location of MLwiN
global MLwiN_path "C:\Program Files\MLwiN v2.25\mlwin.exe"

*Generate the spline variables, in this example 5 new variables are created: s1 (age between birth and 3 months), s2 (age between 3 and 12 months), s3 (age between 12 and 36 months), s4 (age between 36 and 84 months and s5 (age above 84 months).
mkspline s1 3 s2 12 s3 36 s4 84 s5 = age_mt

*MLwiN does not automatically include a constant term, so this must be generated and included in models.
gen cons=1

*generate a new variable that identifies the ordering of observations within individuals
sort id age_mt
by id: gen occ = _n

* generate spline*gender interactions
foreach var of varlist s1 s2 s3 s4 s5 {
gen male`var' = male*`var'
}

*Run the multilevel model, sorting the data by person and occasion first.

*Initial model with no covariates or complex level 1 variation
sort id occ
runmlwin weight cons  s1 s2 s3 s4 s5  ///
        , level2 (id: cons s1 s2 s3 s4 s5, reset(none)) ///
        level1 (occ: cons  , reset(none)) ///
        nopause maxiter(100)

*Adding in gender as a covariate
sort id occ
runmlwin weight cons  s1 s2 s3 s4 s5  ///
        male males1 males2 males3 males4 males5  ///
```

```
    , level2 (id: cons s1 s2 s3 s4 s5, reset(none)) ///
    level1 (occ: cons  ,  reset(none)) ///
    nopause maxiter(100)


*Adding complex level 1 variation and asking Stata to export the level 1 and 2 residuals
sort id occ
runmlwin weight cons  s1 s2 s3 s4 s5  ///
    male males1 males2 males3 males4 males5  ///
    , level2 (id: cons s1 s2 s3 s4 s5, reset(none) residuals(lev2,var)) ///
    level1 (occ: cons  age_mt , diagonal reset(none) residuals(lev1,var)) ///
    nopause maxiter(100)
```

Supplementary Figure 1: Histograms of ages at growth measurements

BiB

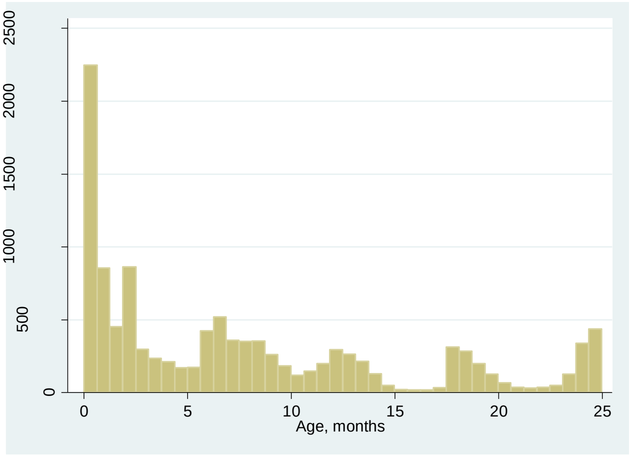

PROBIT

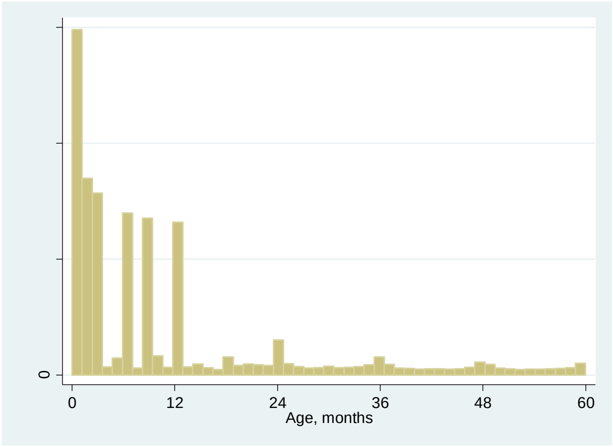

Generation XXI

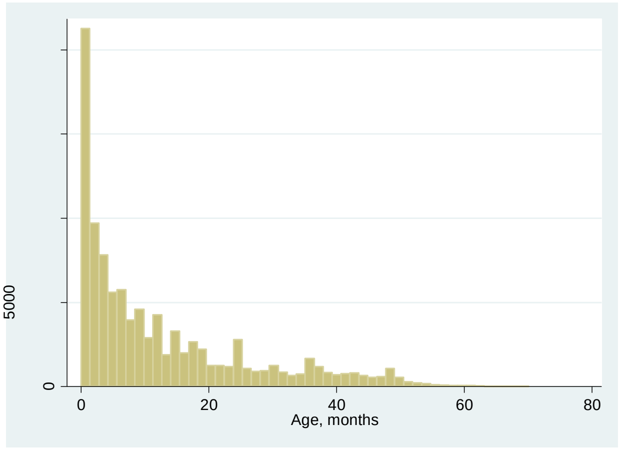

Supplementary Figure 2: Fractional polynomial curves for males' length/height and weight in ALSPAC

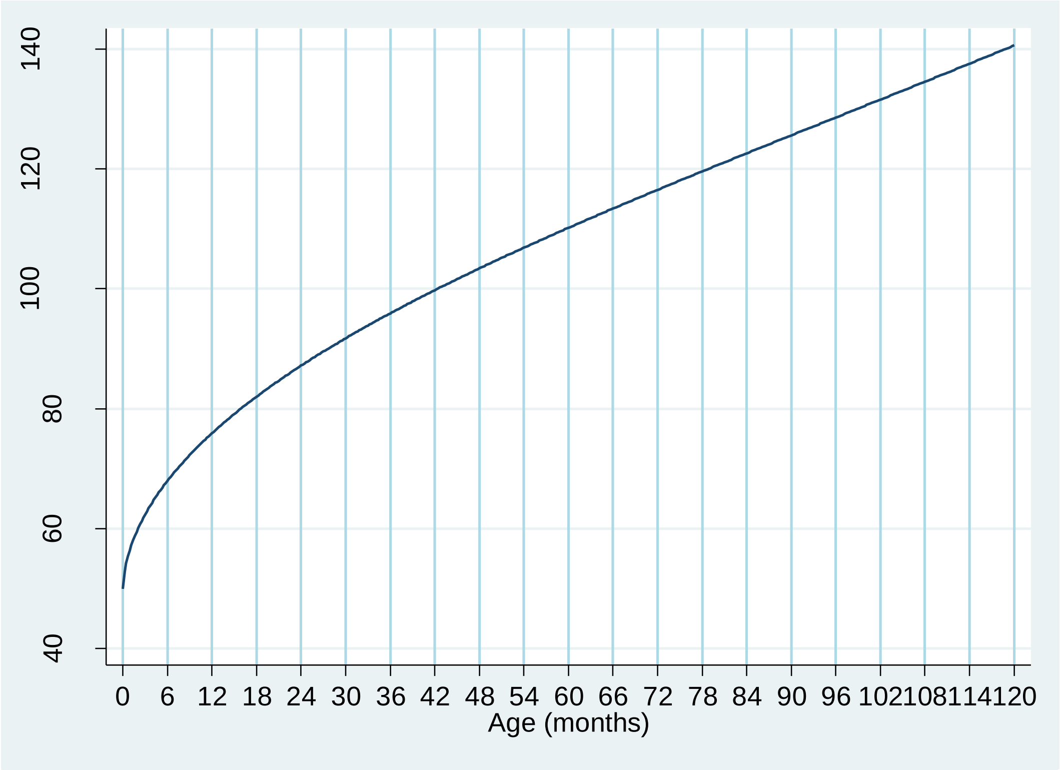

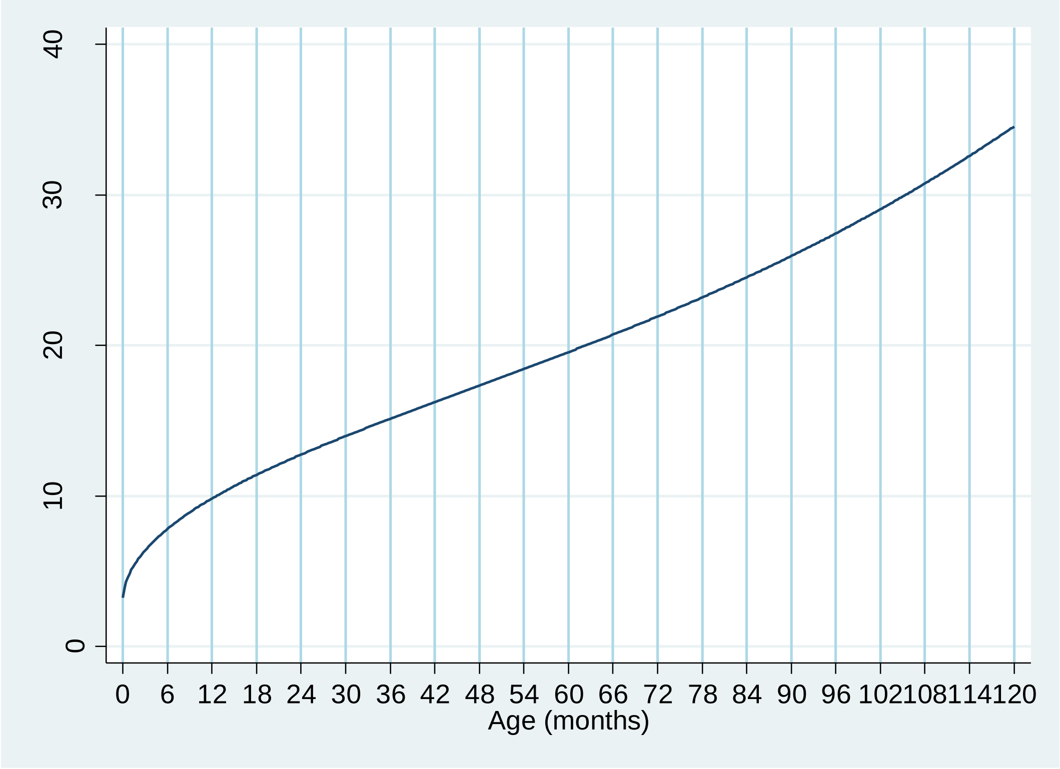

Supplementary Figure 3: Normal plots of residuals for ALSPAC, BiB, Generation XXI, the Pelotas 2004 cohort and PROBIT

a1. ALSPAC weight models:

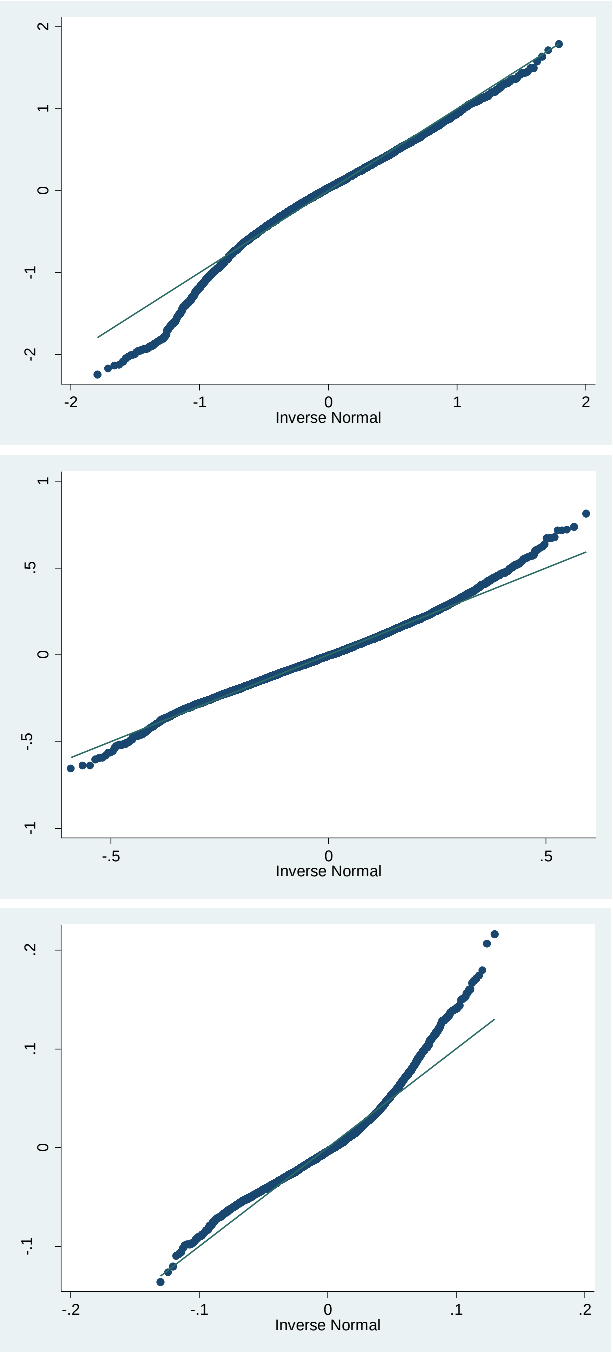

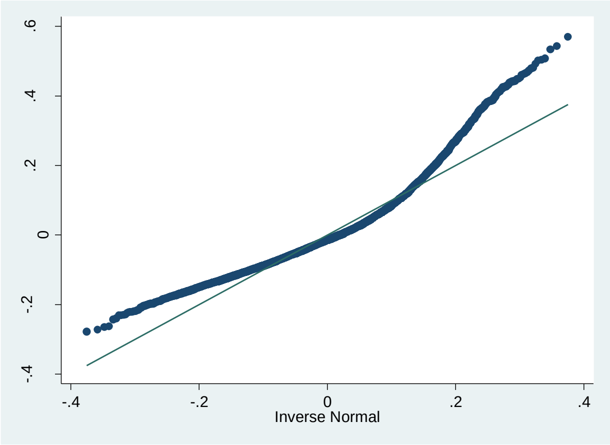
.6
.4
.2
0
-.2
-.4
-.4
-.2
0
.2
.4
Inverse Normal

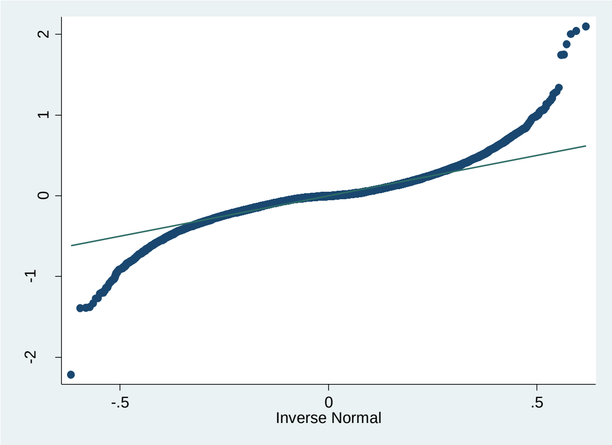
2
1
0
-1
-2
-.5
0
.5
Inverse Normal

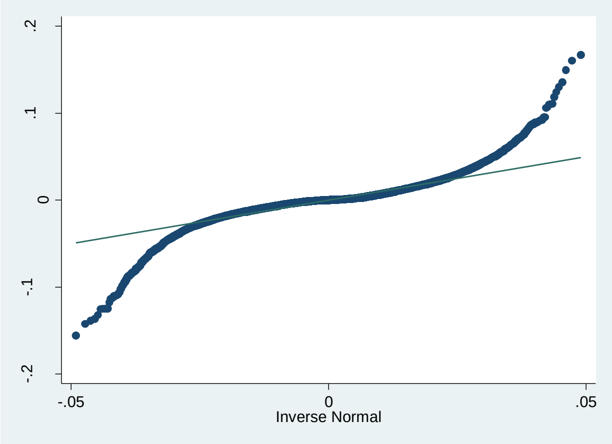
.2
.1
0
-.1
-.2
-.05
0
.05
Inverse Normal

a2. ALSPAC length/height models:

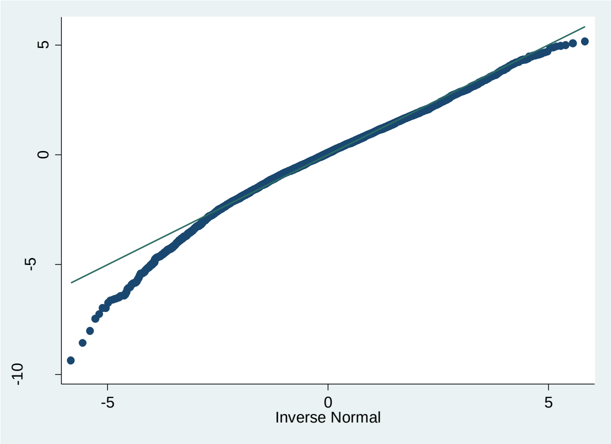

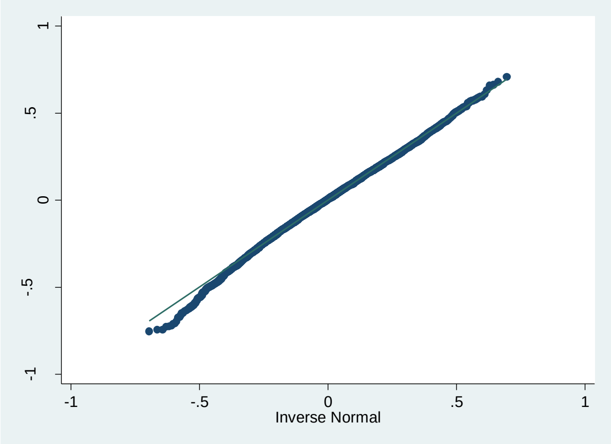

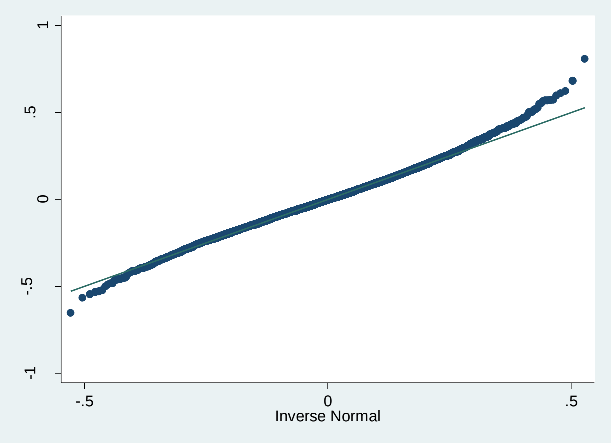

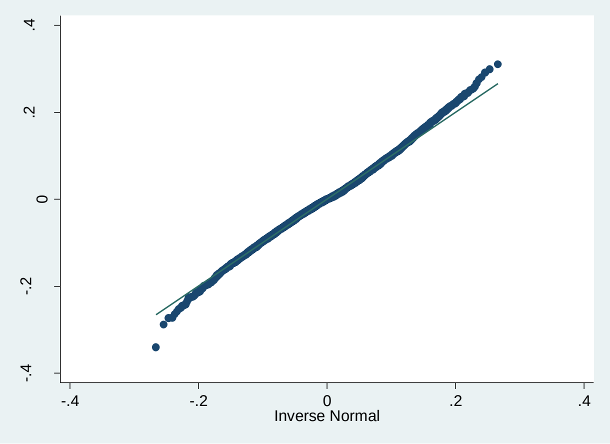

.4
.2
0
-.2
-.4
-.4
-.2
0
.2
.4
Inverse Normal

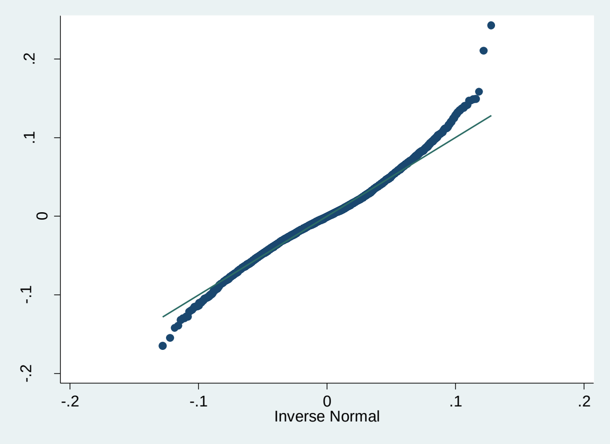

.2
.1
0
-.1
-.2
-.2
-.1
0
.1
.2
Inverse Normal

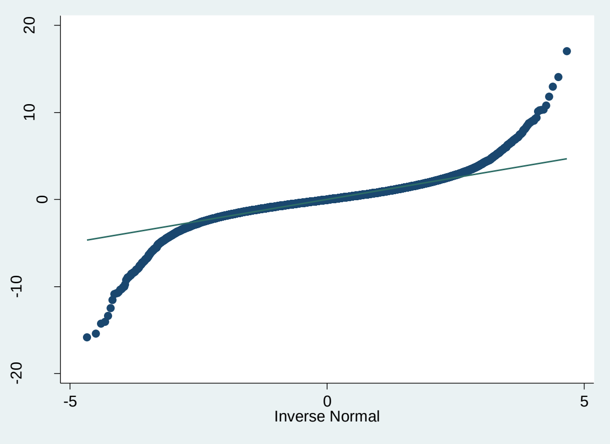

20
10
0
-10
-20
-5
0
5
Inverse Normal

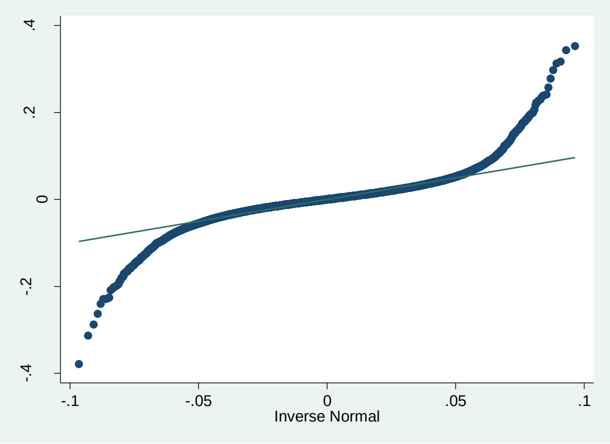

.4
.2
0
-.2
-.4
-.1
-.05
0
.05
.1
Inverse Normal

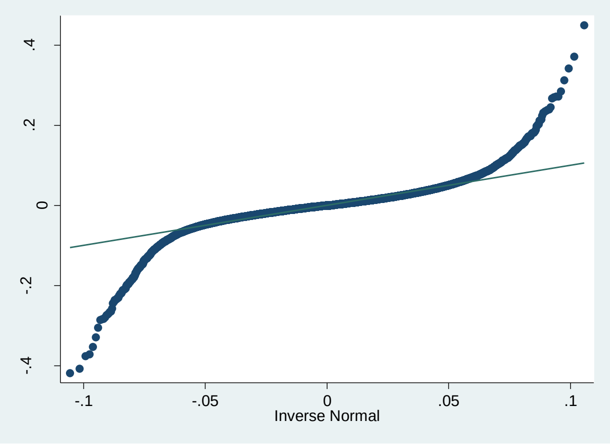
.4
.2
0
-.2
-.4
-.1
-.05
0
.05
.1
Inverse Normal

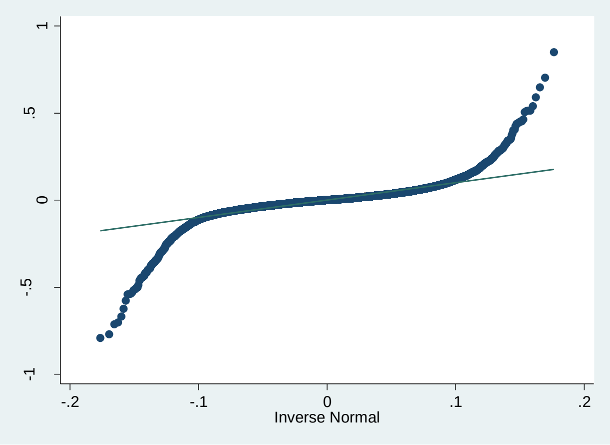
1
.5
0
-.5
-1
-.2
-.1
0
.1
.2
Inverse Normal

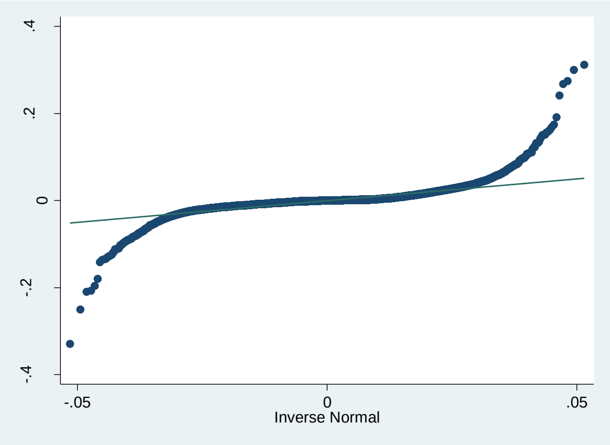
.4
.2
0
-.2
-.4
-.05
0
.05
Inverse Normal

b1. BiB weight models:

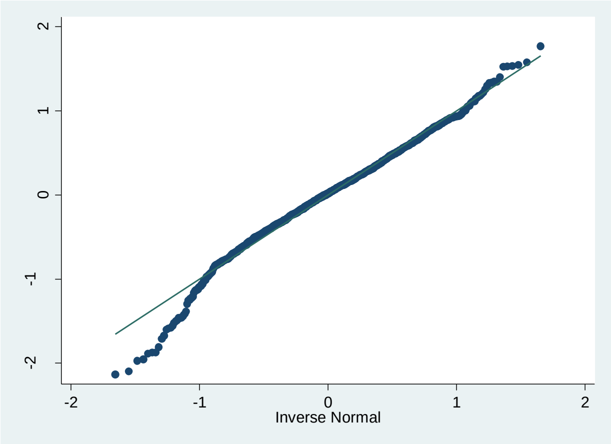

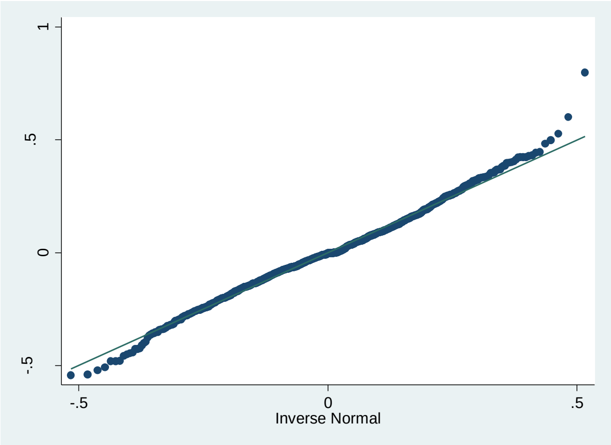

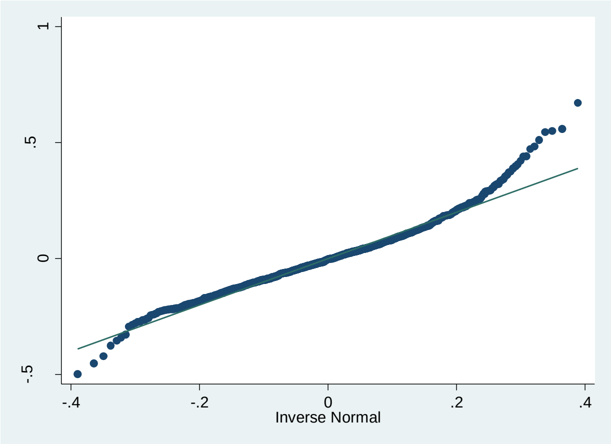

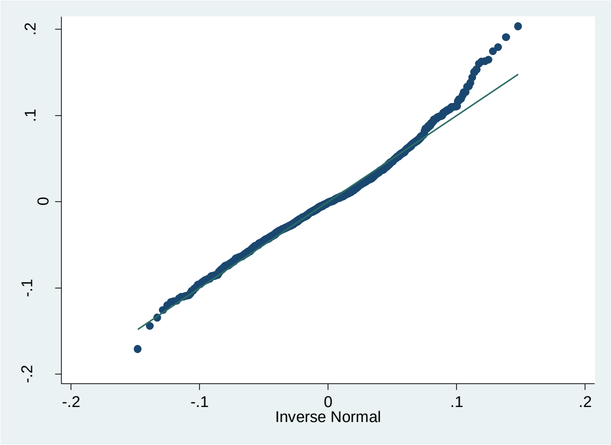
.2
.1
0
-.1
-.2
-.2
-.1
0
.1
.2
Inverse Normal

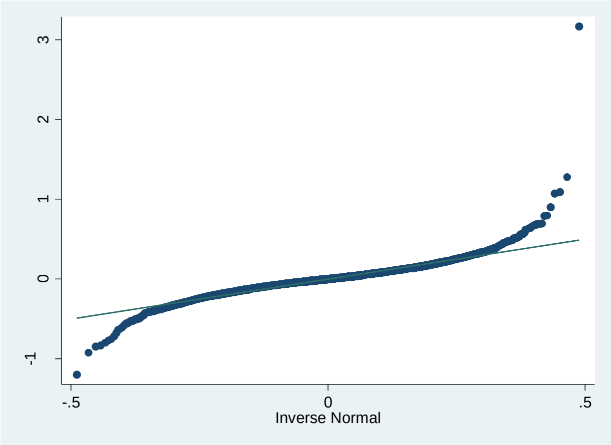
3
2
1
0
-1
-.5
0
.5
Inverse Normal

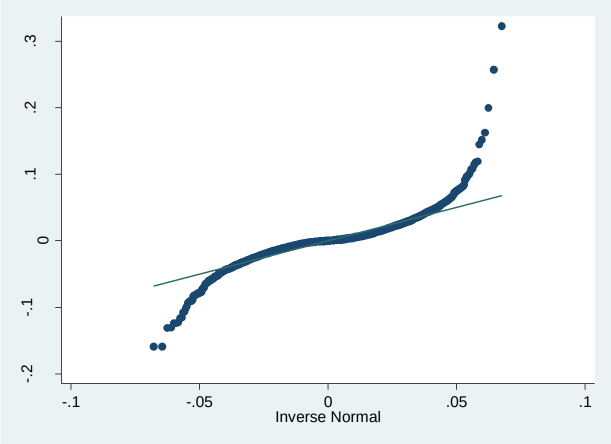
.3
.2
.1
0
-.1
-.2
-.1
-.05
0
.05
.1
Inverse Normal

b2. BiB length/height models:

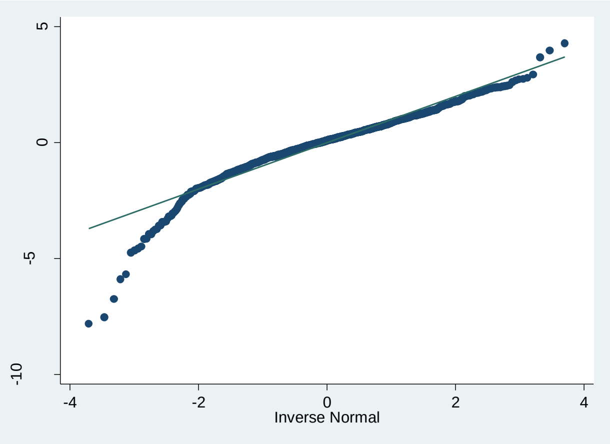

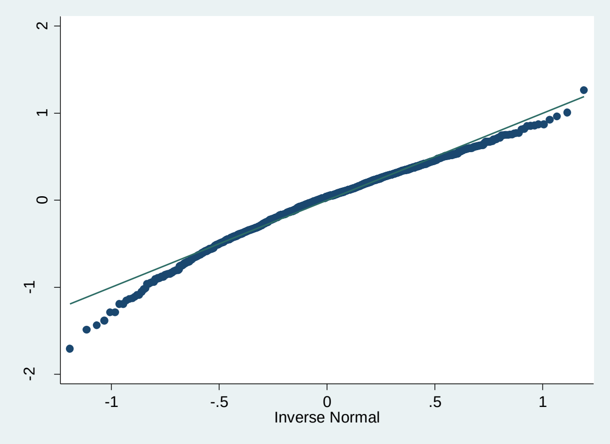

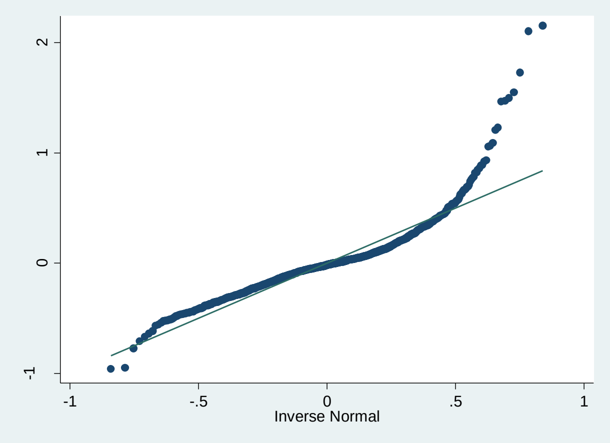

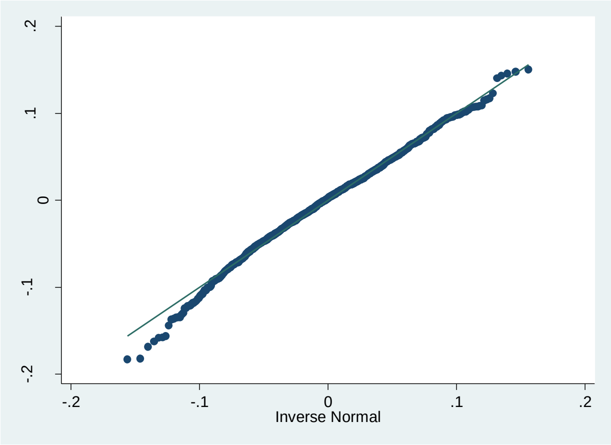

.2
.1
0
-.1
-.2
-.2
-.1
0
.1
.2
Inverse Normal

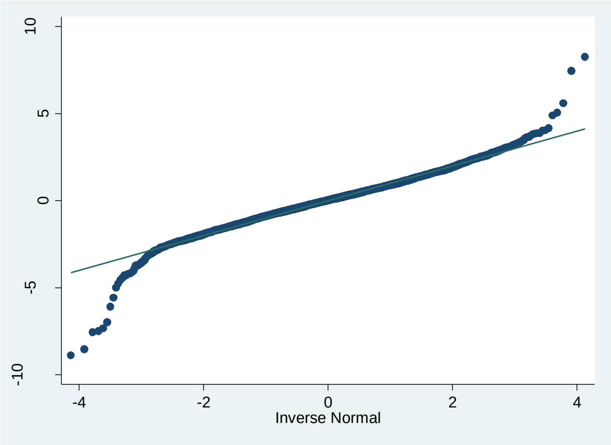

10
5
0
-5
-10
-4
-2
0
2
4
Inverse Normal

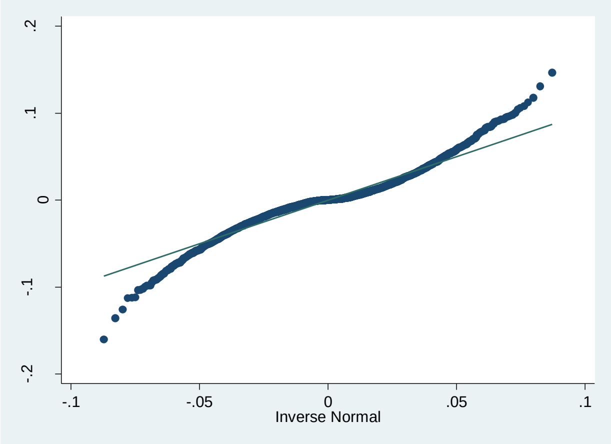

.2
.1
0
-.1
-.2
-.1
-.05
0
.05
.1
Inverse Normal

c1. Generation XXI weight models:

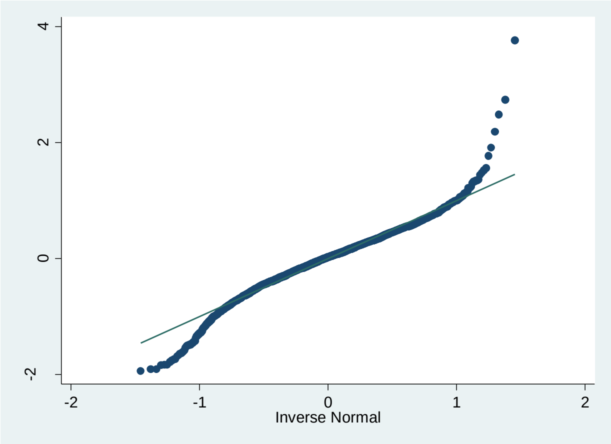

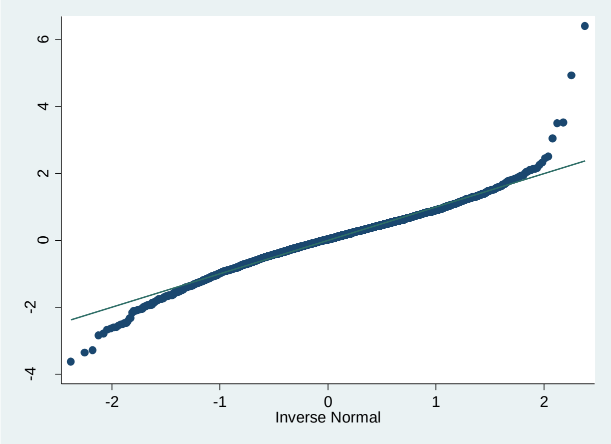

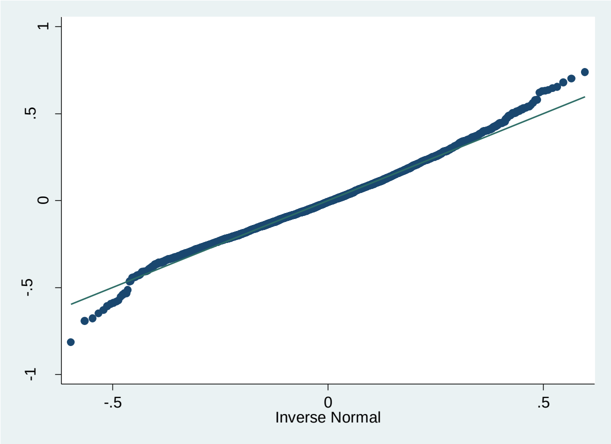

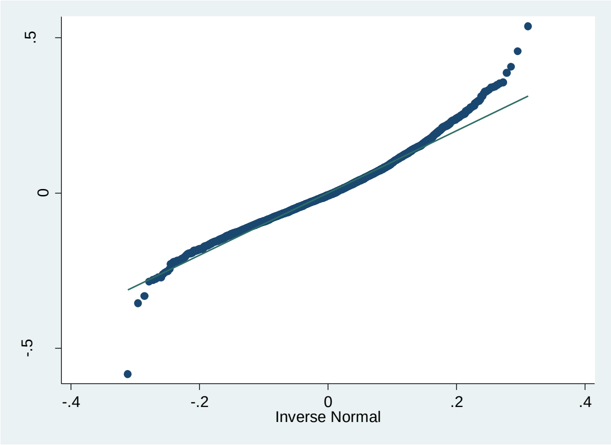
.5
0
-.5
-.4
-.2
0
.2
.4
Inverse Normal

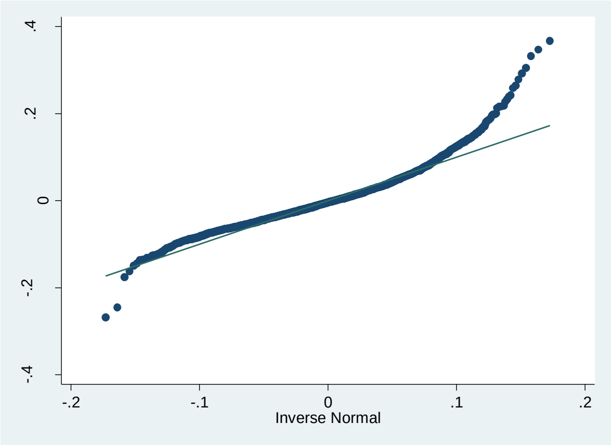
.4
.2
0
-.2
-.4
-.2
-.1
0
.1
.2
Inverse Normal

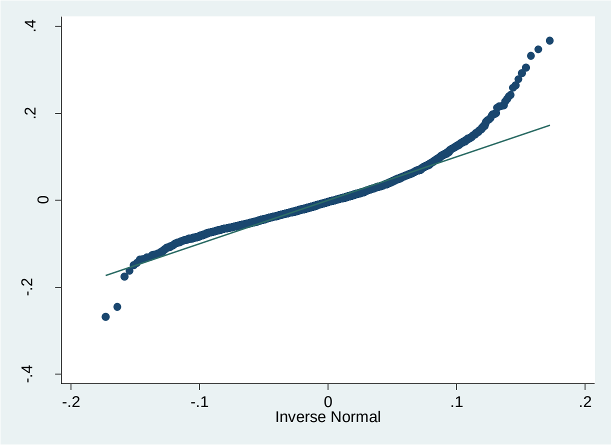
.4
.2
0
-.2
-.4
-.2
-.1
0
.1
.2
Inverse Normal

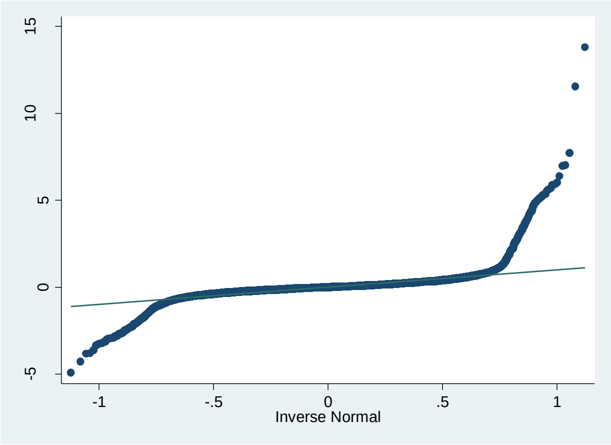

15
10
5
0
-5
-1
-.5
0
.5
1
Inverse Normal

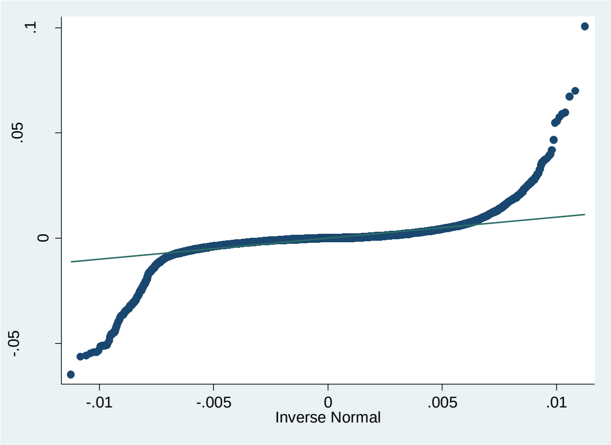

.1
.05
0
-.05
-.01
-.005
0
.005
.01
Inverse Normal

c2. Generation XXI length/height models:

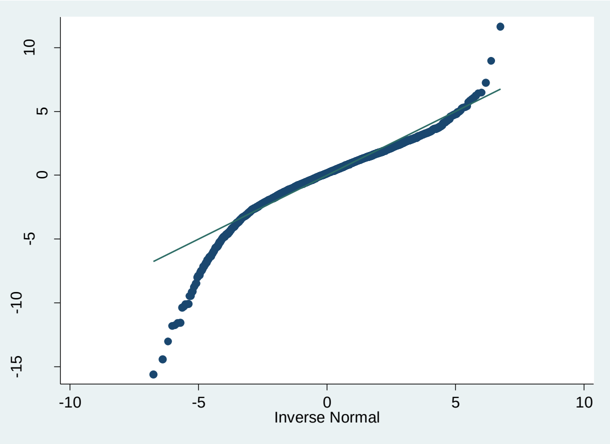

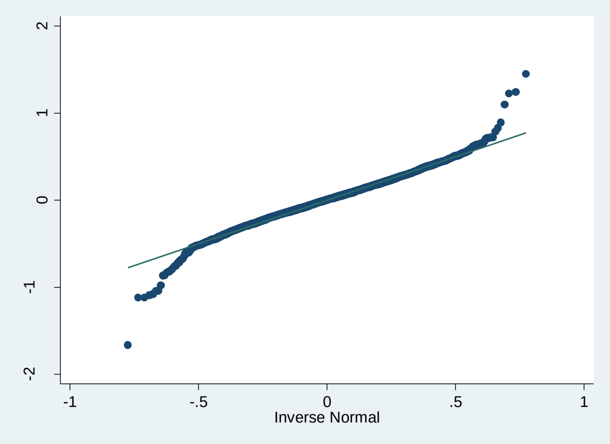

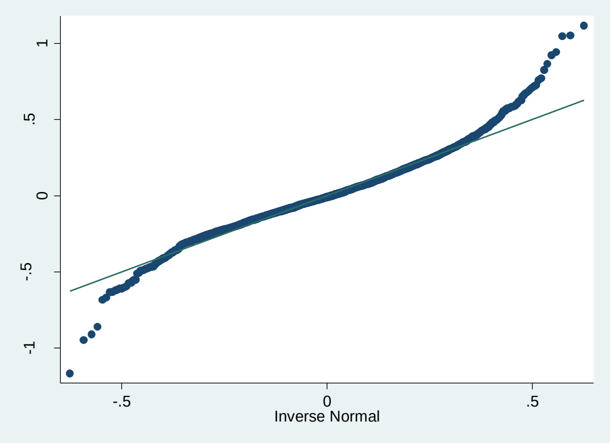

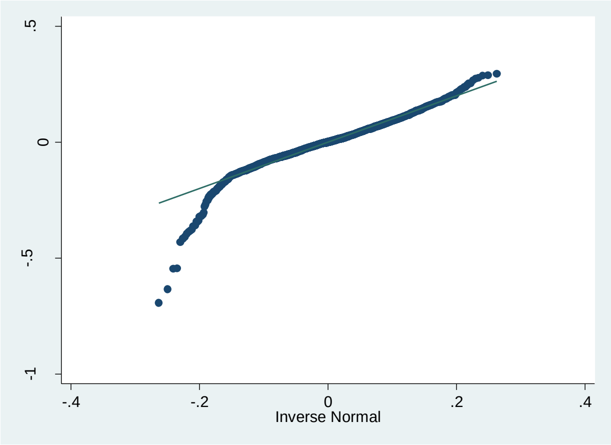
.5
0
-.5
-1
-.4
-.2
0
.2
.4
Inverse Normal

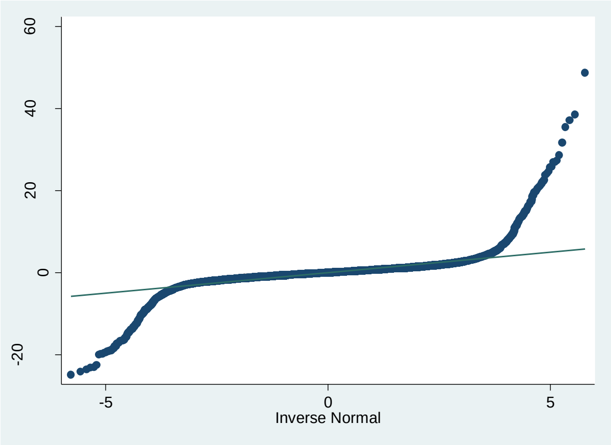
60
40
20
0
-20
-5
0
5
Inverse Normal

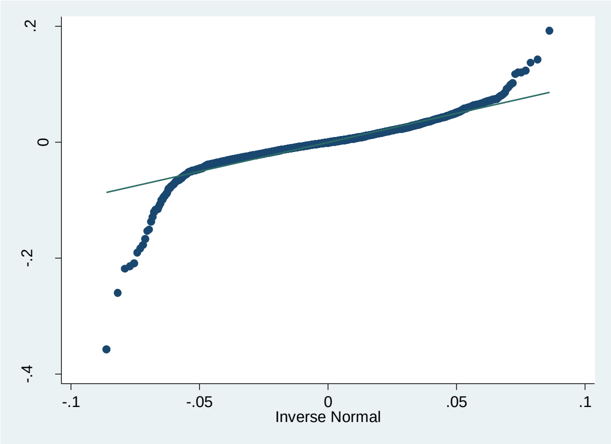
.2
0
-.2
-.4
-.1
-.05
0
.05
.1
Inverse Normal

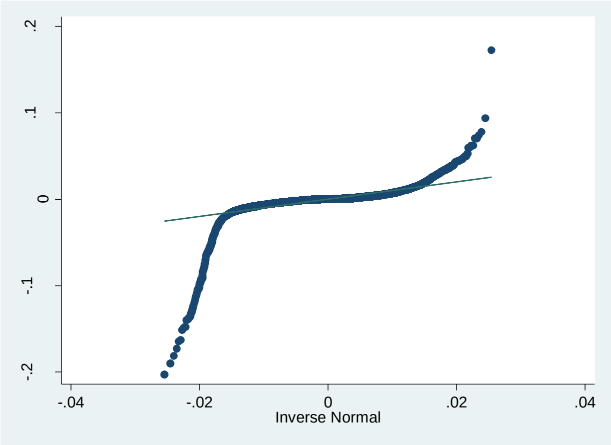
.2
.1
0
-.1
-.2
-.04
-.02
0
.02
.04
Inverse Normal

d1. Pelotas 2004 weight models:

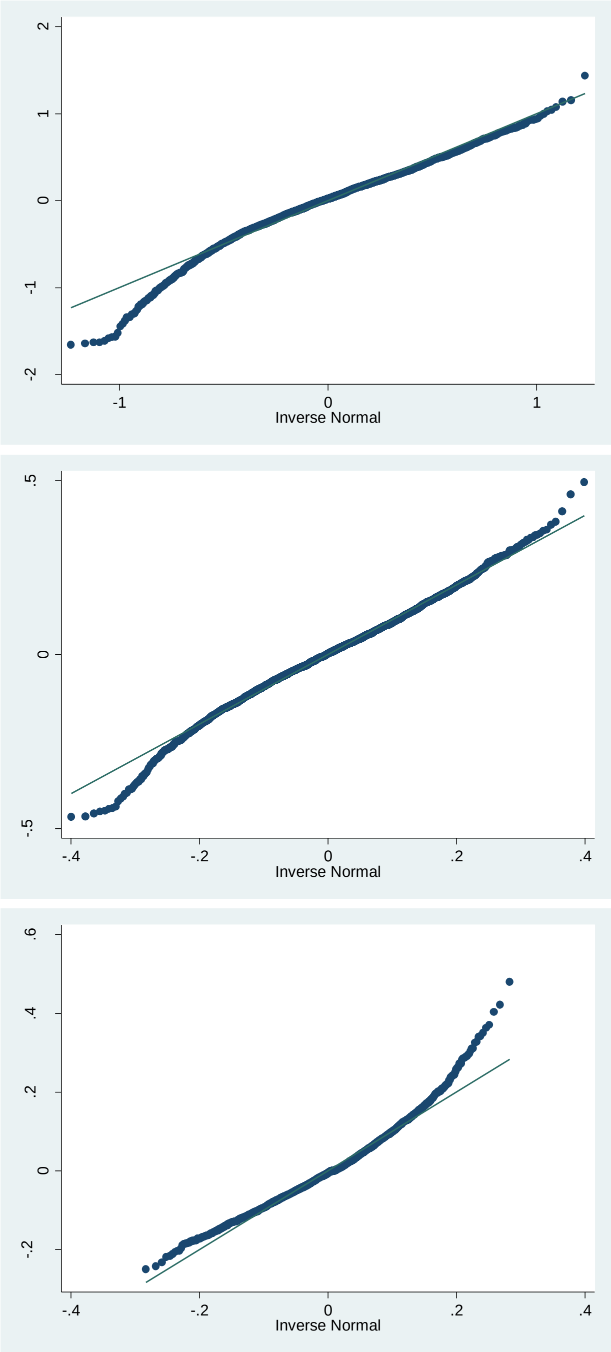

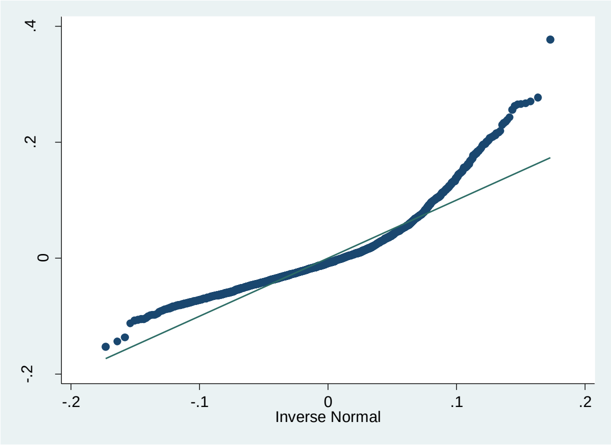

.4
.2
0
-.2
-.2
-.1
0
.1
.2
Inverse Normal

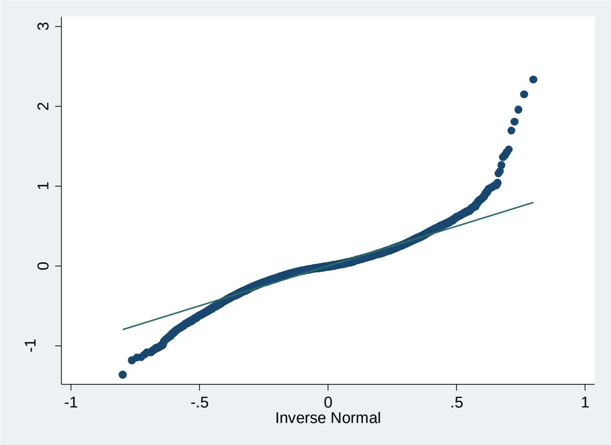

3
2
1
0
-1
-1
-.5
0
.5
1
Inverse Normal

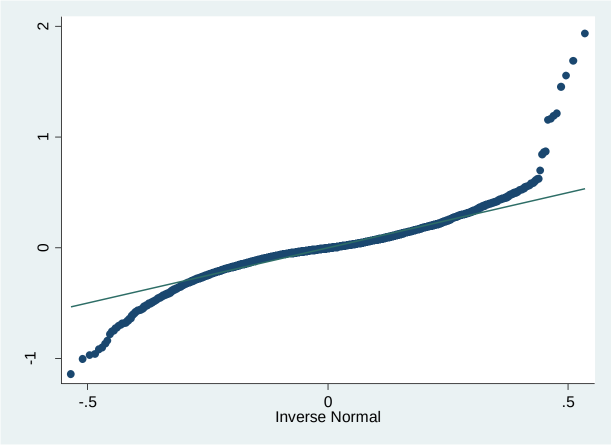

2
1
0
-1
-.5
0
.5
Inverse Normal

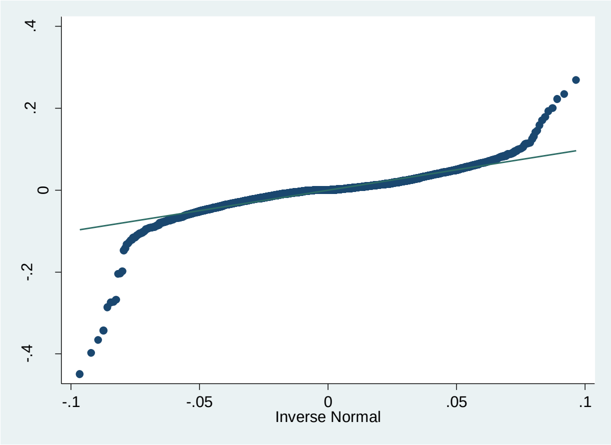
.4
.2
0
-.2
-.4
-.1
-.05
0
.05
.1
Inverse Normal

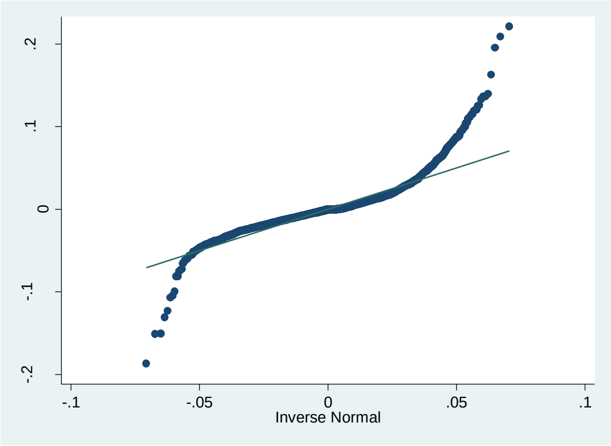
.2
.1
0
-.1
-.2
-.1
-.05
0
.05
.1
Inverse Normal

d2. Pelotas 2004 length/height models:

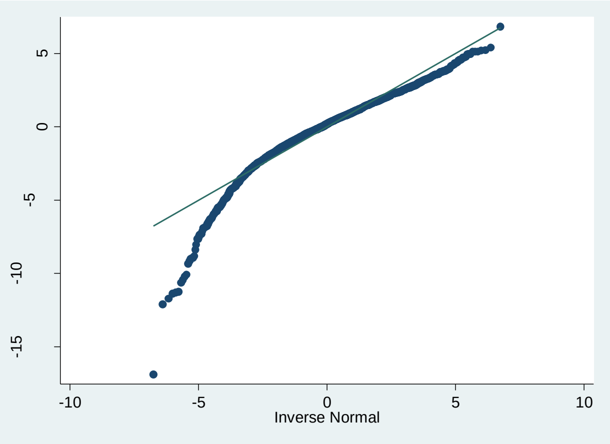

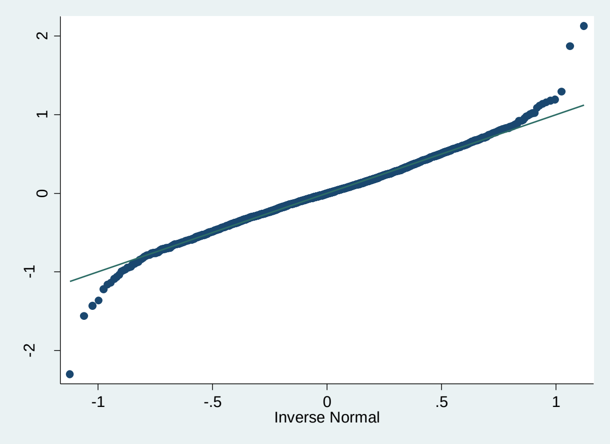

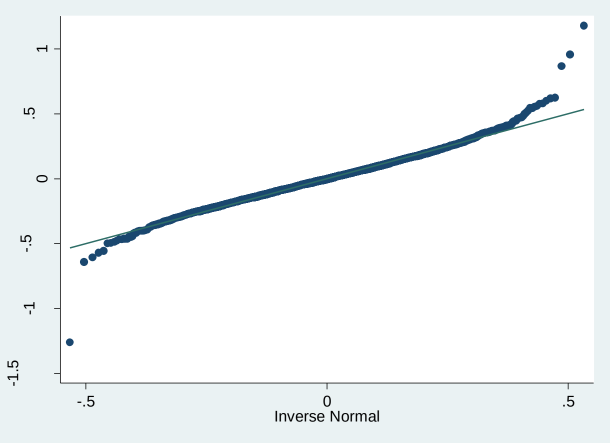

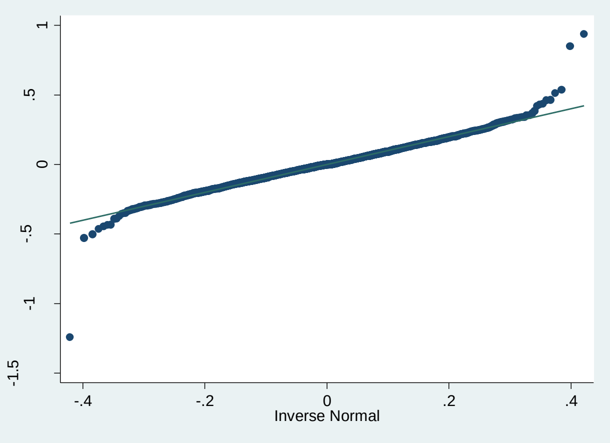
1
.5
0
-.5
-1
-1.5
-.4
-.2
0
.2
.4
Inverse Normal

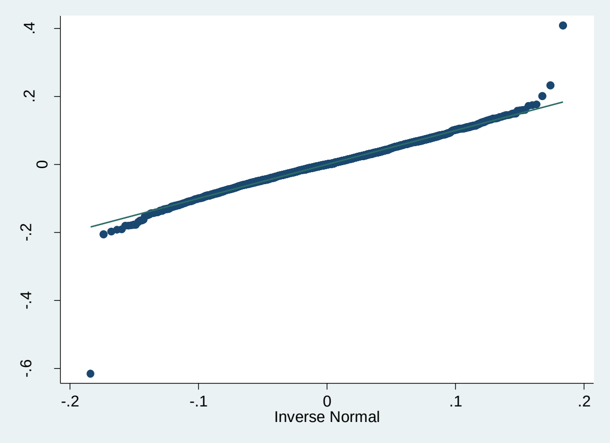
.4
.2
0
-.2
-.4
-.6
-.2
-.1
0
.1
.2
Inverse Normal

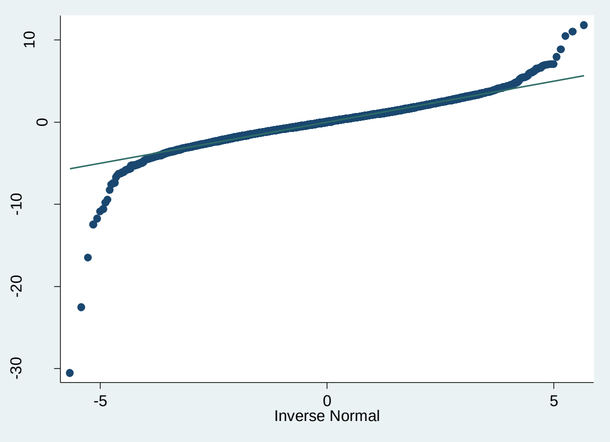
10
0
-10
-20
-30
-5
0
5
Inverse Normal

e1. PROBIT weight models:

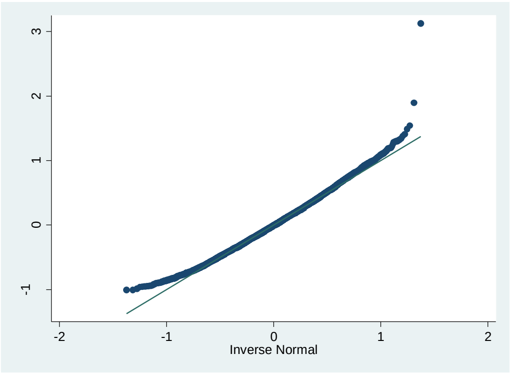

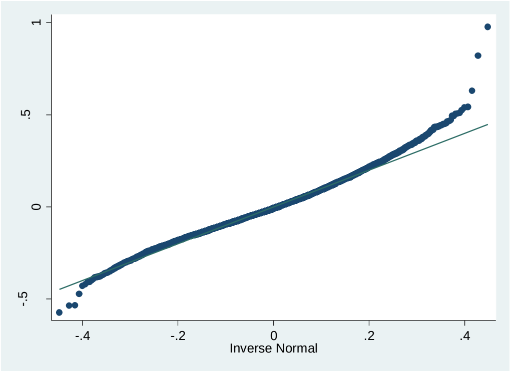

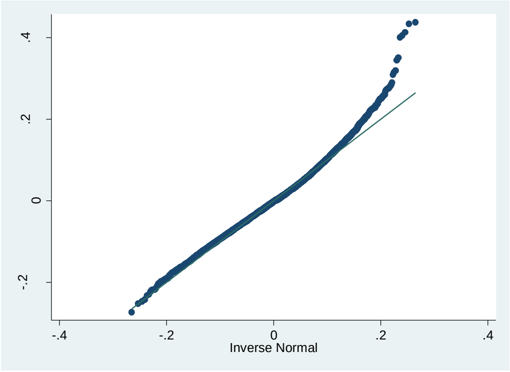

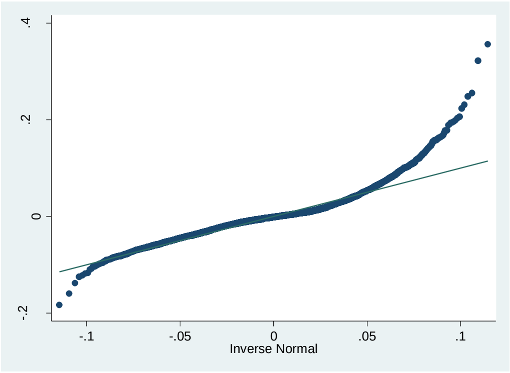

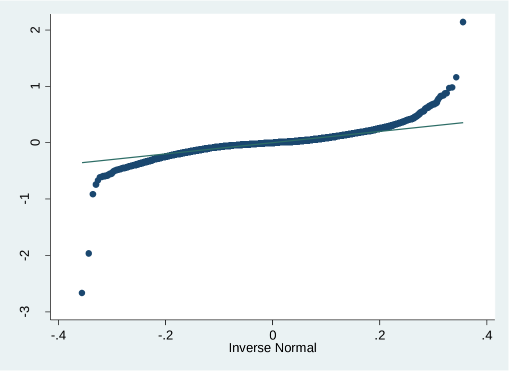
-3
-2
-1
0
1
2
-.4
-.2
0
.2
.4
Inverse Normal

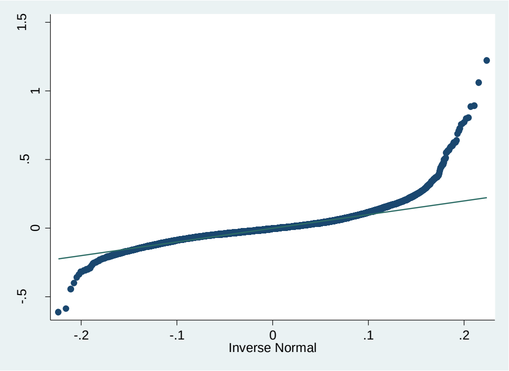
-.5
0
.5
1
1.5
-.2
-.1
0
.1
.2
Inverse Normal

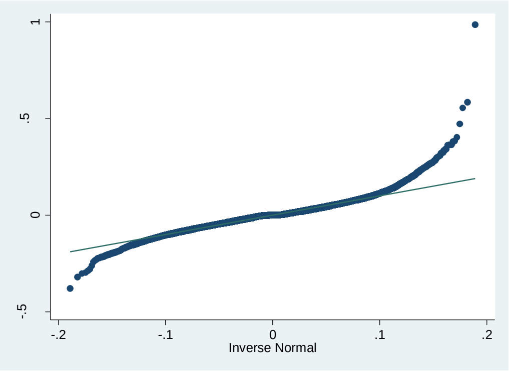
-.5
0
.5
1
-.2
-.1
0
.1
.2
Inverse Normal

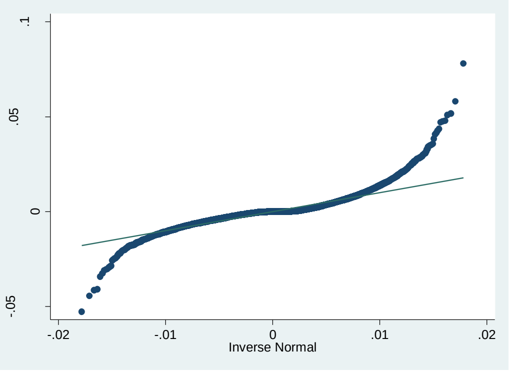
-.05
0
.05
.1
-.02
-.01
0
.01
.02
Inverse Normal

e2. PROBIT length/height models:

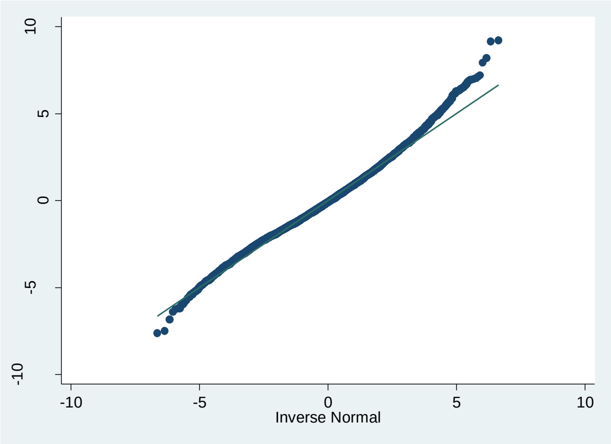

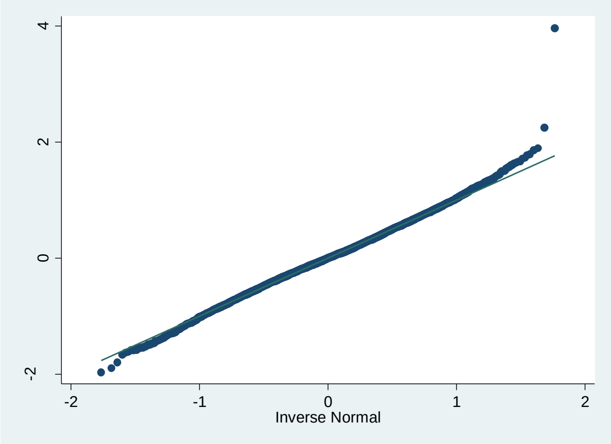

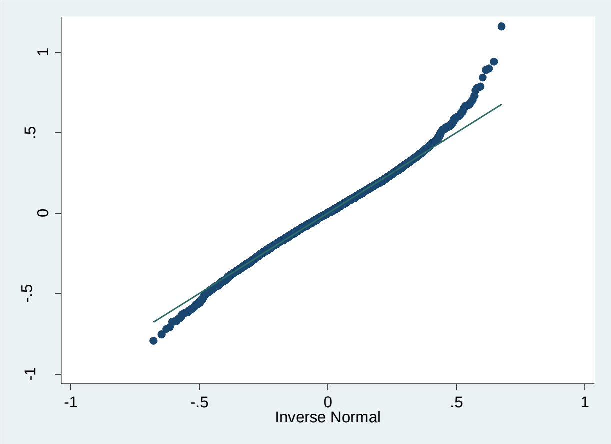

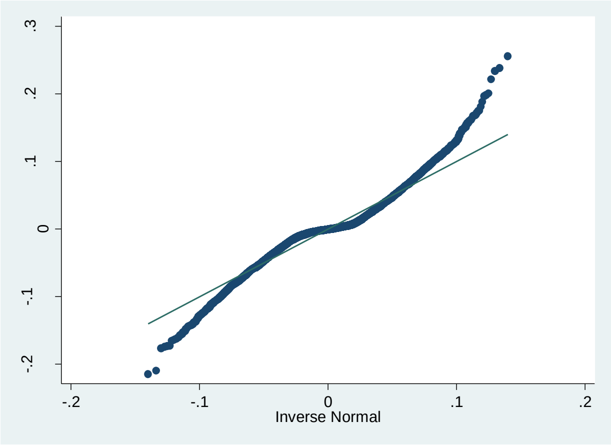
.3
.2
.1
0
-.1
-.2
-.2
-.1
0
.1
.2
Inverse Normal

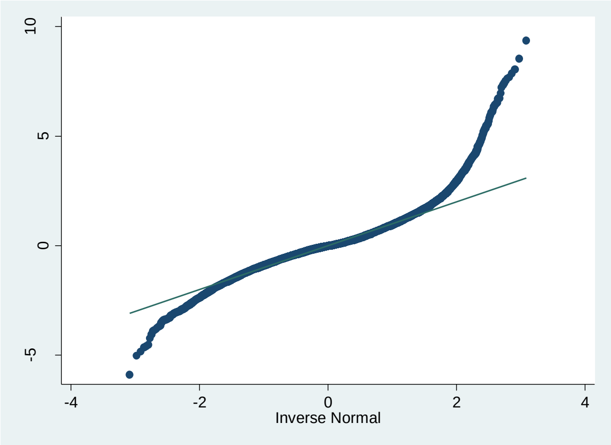
10
5
0
-5
-4
-2
0
2
4
Inverse Normal

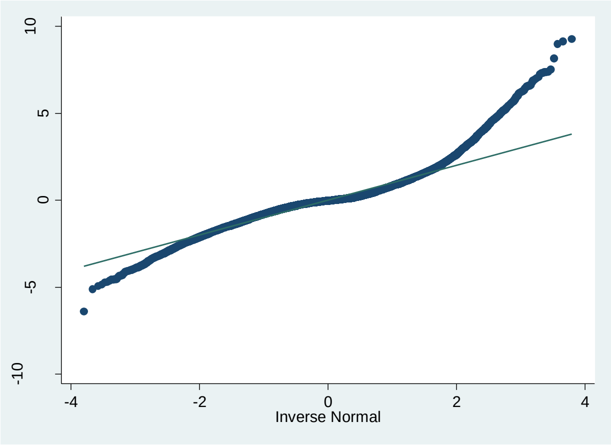
10
5
0
-5
-10
-4
-2
0
2
4
Inverse Normal

Supplementary Figure 4: Correlations between successive level 1 residuals

The residuals from a linear regression of level-1 residuals on the previous level-1 residual for the same individual are plotted against the time difference between the consecutive measures

a1. ALSPAC weight model

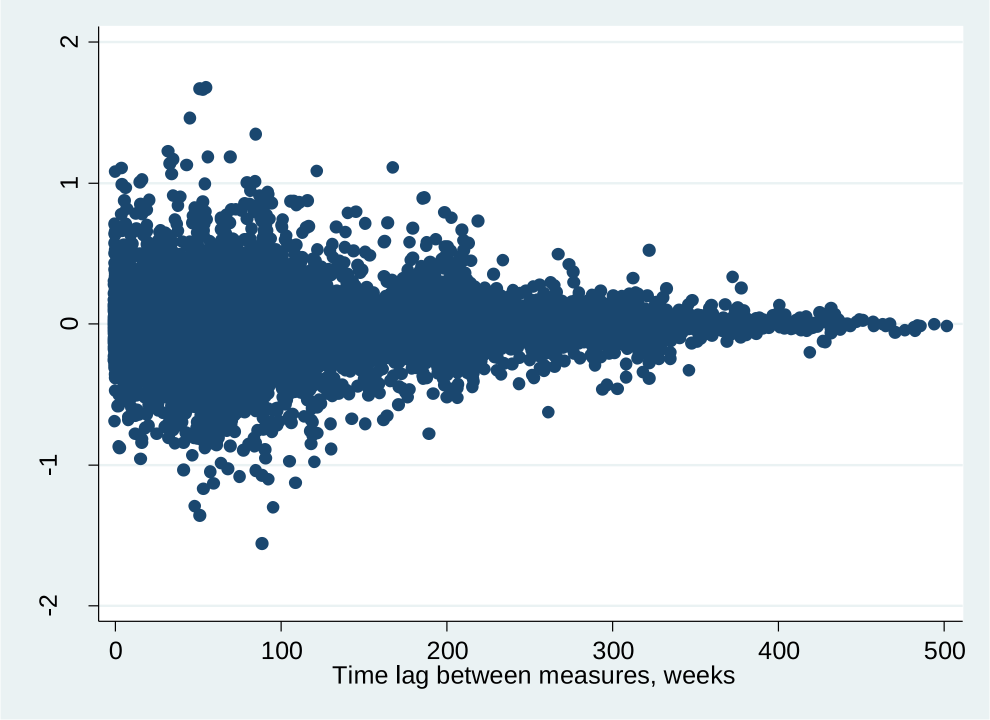

a2. ALSPAC length/height model

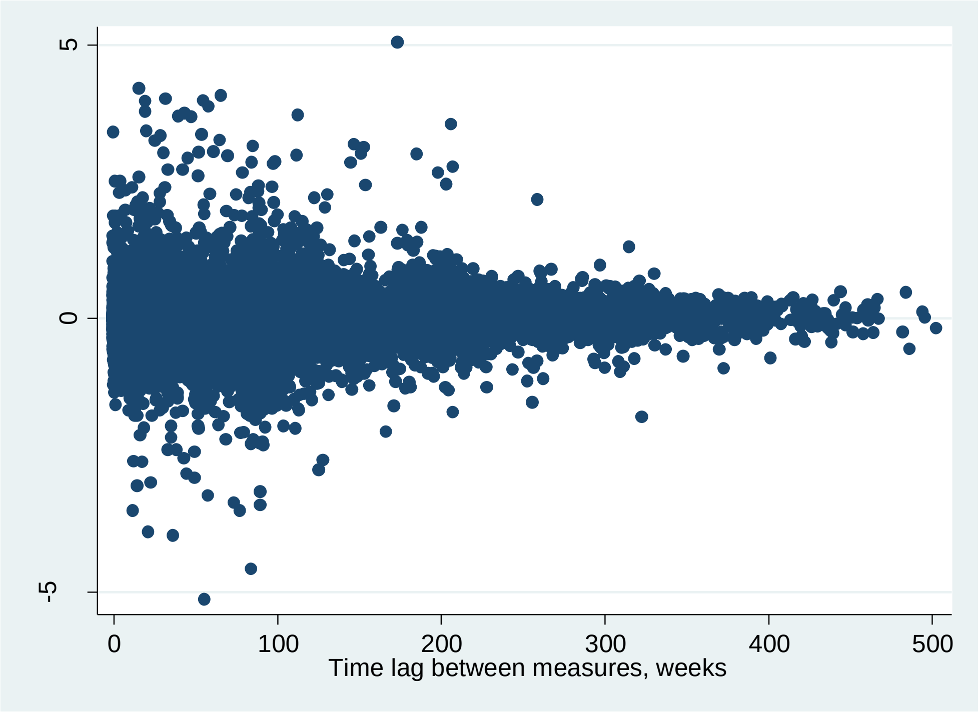

b1. BiB weight model

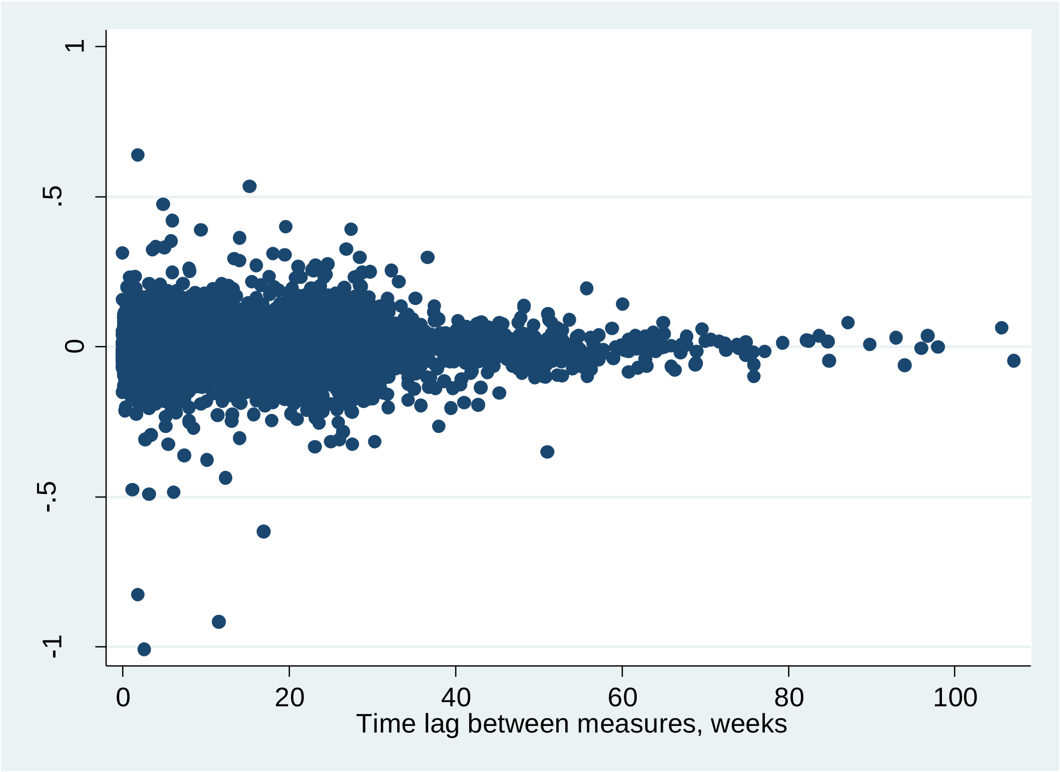

b2. BiB length/height model

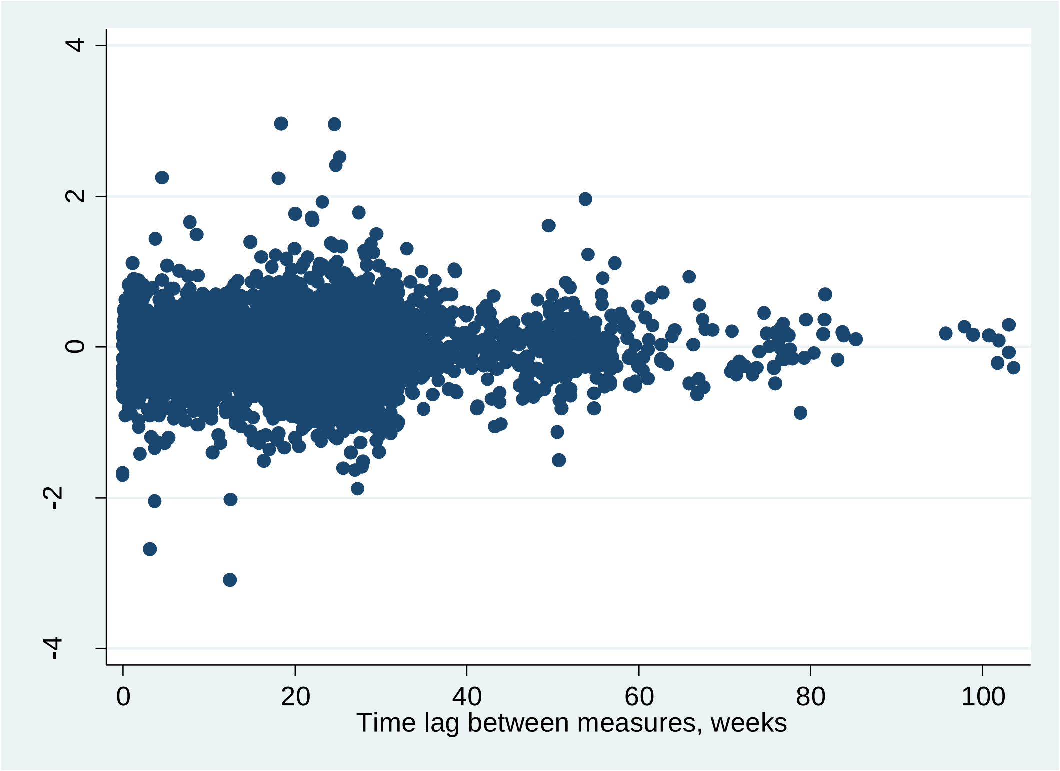

c1. Generation XXI weight model

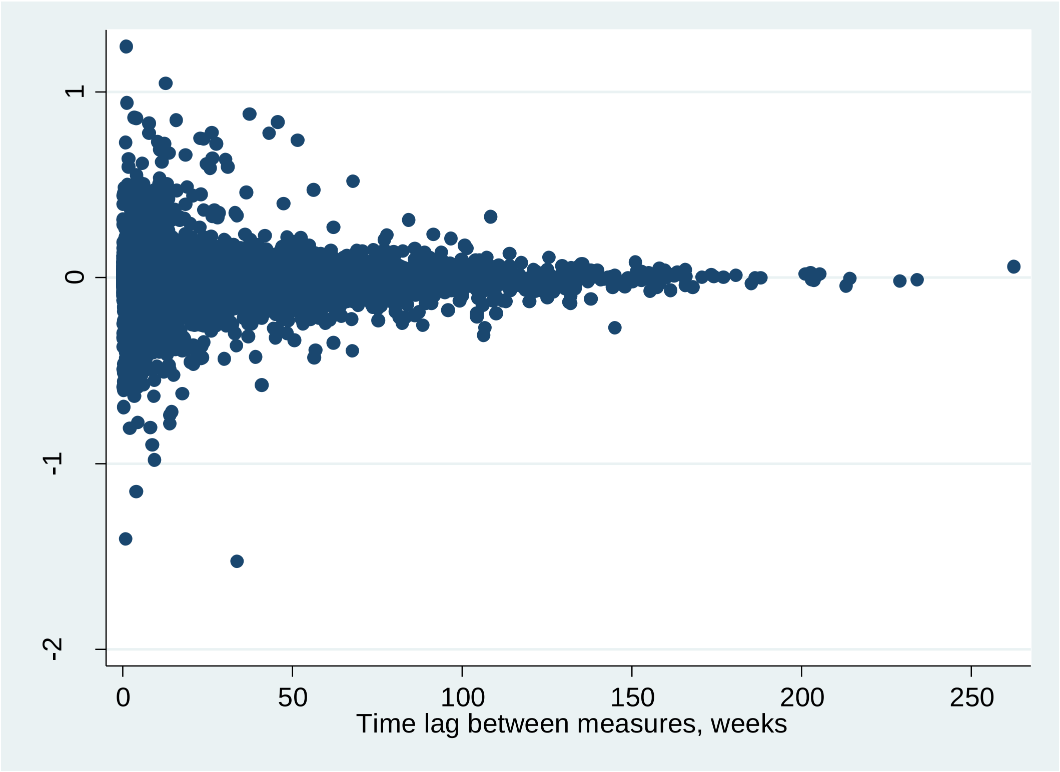

c2. Generation XXI length/height model

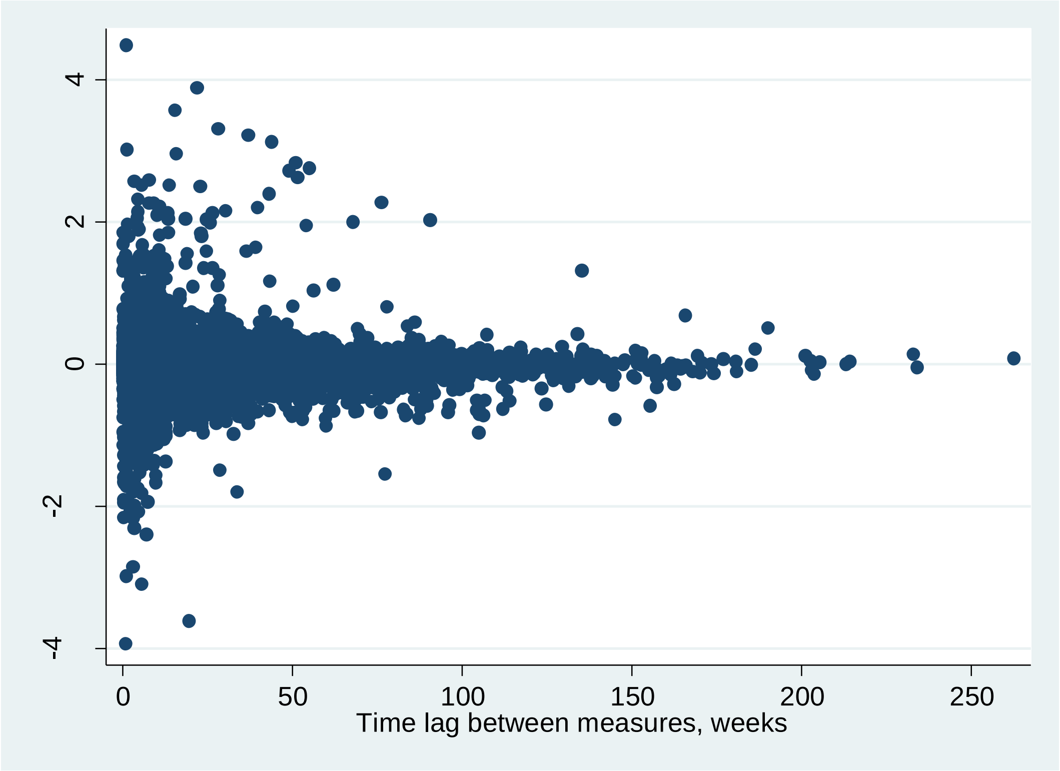

d1. Pelotas weight model

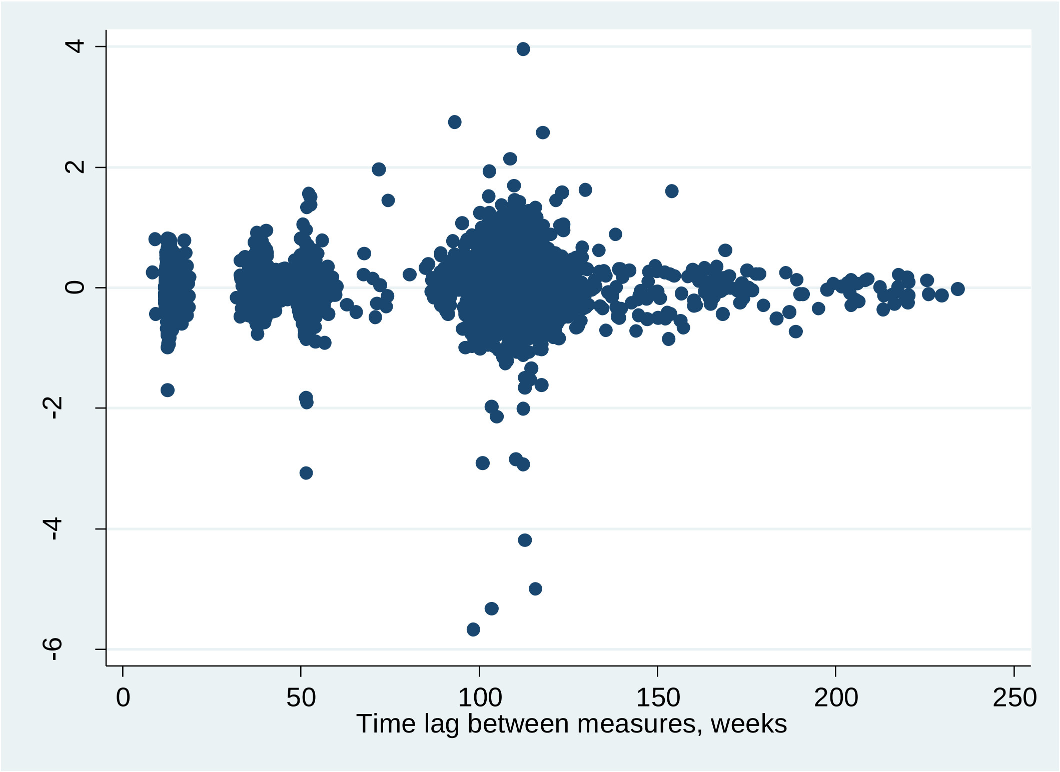

d2. Pelotas length/height model

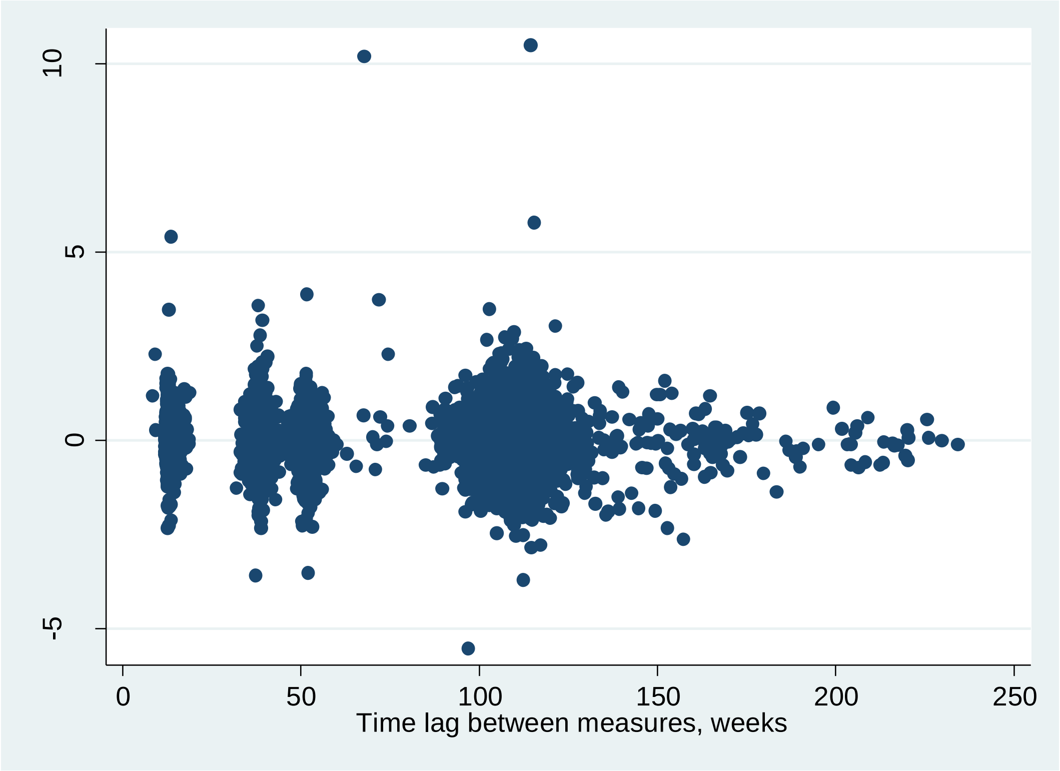

e1. PROBIT weight model

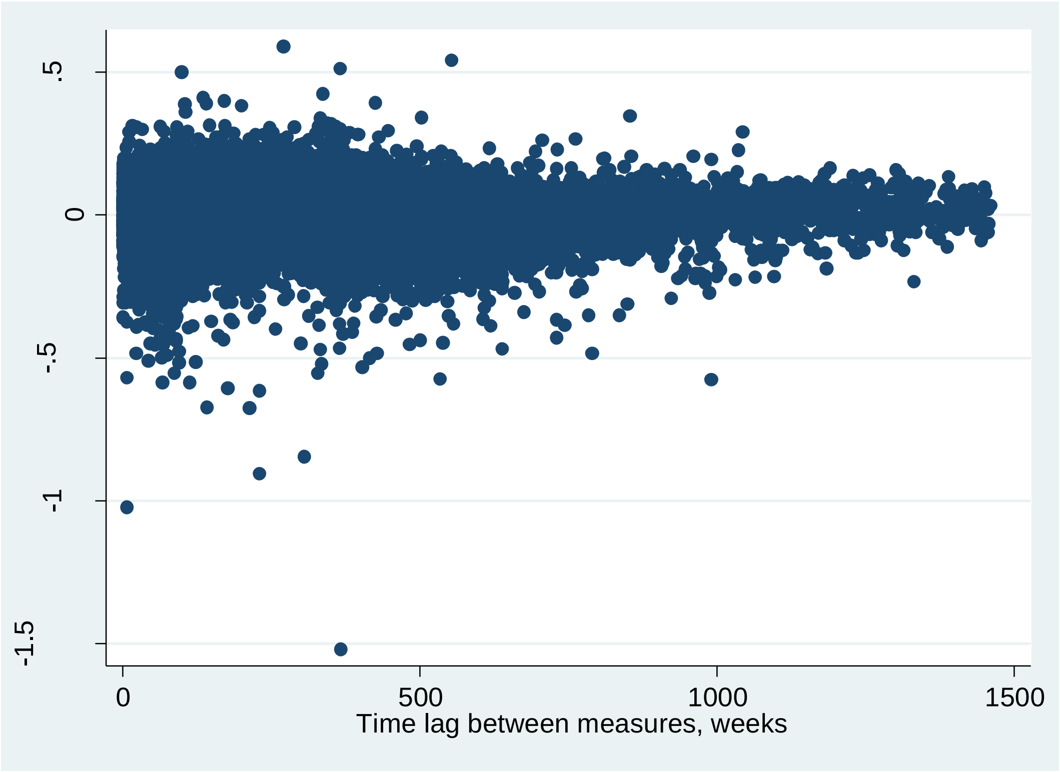

e2. PROBIT length/height model

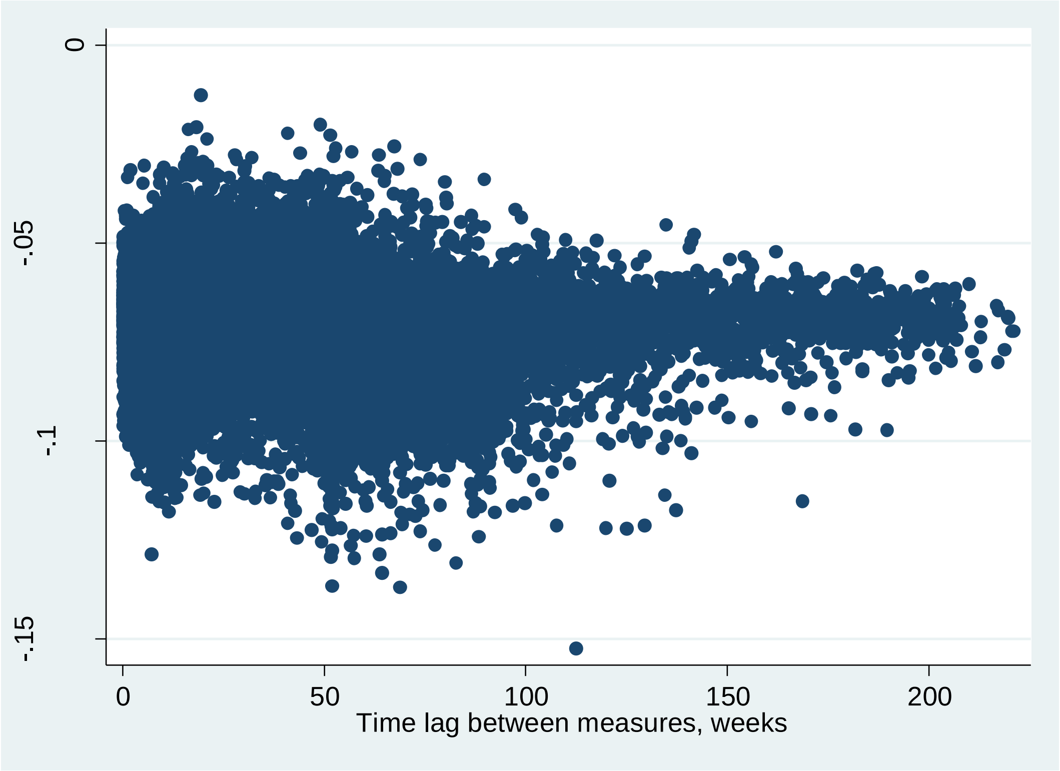

Supplementary Table 1: Difference in model fit for the ALSPAC growth trajectories using the best-fitting knot points defined by deviance, and knot points placed at whole years or fractions of years

	Mean observed measurement (SD)	Mean difference between observed and predicted	95% reference range for the differences
ALSPAC models using best-fitting knot points defined by deviance			
Males			
Length/height models:			
Birth length	50.97 (2.39)	-0.10	-2.14 to 1.93
>0 to ≤3 months	57.85 (3.15)	0.056	-2.35 to 2.25
>3 to ≤10 months	70.22 (4.65)	-0.11	-2.52 to 2.30
>10 to ≤29 months	82.42 (5.43)	-0.0050	-2.77 to 2.76
>29 months to ≤10 years	116.98 (15.65)	0.017	-3.35 to 3.39
Weight models:			
Birth weight	3.44 (0.58)	-0.0041	-0.34 to 0.33
>0 to ≤4 months	4.95 (1.10)	0.0086	-0.36 to 0.38
>4 to ≤11 months	9.04 (1.28)	-0.033	-0.55 to 0.48
>11 to ≤80 months	15.38 (4.24)	0.031	-1.50 to 1.56
>80 months to ≤10 years	29.28 (6.30)	-0.058	-2.10 to 1.98
ALSPAC models using best-fitting knot points defined by deviance			
Females			
Length/height models:			
Birth length	50.22 (2.26)	-0.012	-2.11 to 2.09
>0 to ≤2 months	56.10 (2.74)	-0.059	-2.37 to 2.25
>2 to ≤11 months	67.19 (5.72)	0.055	-2.45 to 2.56
>11 to ≤32 months	82.21 (5.65)	-0.12	-2.98 to 2.74
>32 months to ≤10 years	117.28 (15.45)	0.018	-3.31 to 3.35
Weight models:			
Birth weight	3.34 (0.52)	0.00092	-0.27 to 0.28
>0 to ≤4 months	4.60 (0.95)	0.00025	-0.31 to 0.31
>4 to ≤10 months	8.33 (1.16)	-0.054	-0.49 to 0.38
>10 to ≤80 months	14.66 (4.37)	0.0065	-1.56 to 1.57
>80 months to ≤10 years	29.50 (6.68)	-0.12	-1.26 to 1.02
ALSPAC models using knot points at whole years or fractions of years			
Males and Females combined			
Length/height models:			
Birth length	50.61 (2.36)	-0.11 (1.19)	-2.44 to 2.22
>0 to ≤3 months	57.25 (3.11)	0.07 (1.33)	-2.54 to 2.68
>3 months to ≤1 year	69.95 (4.78)	-0.11 (1.38)	-2.81 to 2.59
>1 year to ≤3 years	83.64 (5.56)	0.02 (1.48)	-2.88 to 2.92
>3 years to ≤10 years	117.70 (15.26)	0.05 (1.71)	-3.30 to 3.40
Weight models:			
Birth weight	3.40 (0.55)	0.04 (0.15)	-0.25 to 0.33
>0 to ≤3 months	4.61 (0.91)	-0.04 (0.17)	-0.37 to 0.29
>3 months to ≤1 year	8.49 (1.48)	0.05 (0.27)	-0.48 to 0.58
>1 year to ≤7 years	15.28 (4.26)	-0.01 (0.79)	-1.56 to 1.54
>7 years to ≤10 years	29.41 (6.48)	-0.01 (1.06)	-2.09 to 2.07

Supplementary Table 2: Summary of the number of individuals and measurements included in the growth models

	Number of children with at least one measurement per growth period	Total number of measurements	Median (IQR) of measurements per child*
ALSPAC, N=13,922			
Length/height models:			
Overall	13,922	106,933	7 (5 to 9)
Birth length	10,456	10,456	1
>0 to ≤3 months	11,721	14,043	1 (1 to 1)
>3 months to ≤1 year	11,386	19,164	1 (1 to 2)
>1 year to ≤3 years	10,415	18,056	1 (1 to 2)
>3 years to ≤10 years	12,468	45,214	3 (1 to 4)
Weight models:			
Overall	13,922	120,081	8 (5 to 11)
Birth weight	13,651	13,651	1
>0 to ≤3 months	13,117	24,053	2 (1 to 2)
>3 months to ≤1 year	12,340	21,584	1 (1 to 2)
>1 year to ≤7 years	13,117	40,770	1 (1 to 2)
>7 years to ≤10 years	8,623	20,023	2 (1 to 3)
Born in Bradford, N=1,390			
Length/height models:			
Overall	1,390	5,472	4 (3 to 5)
Birth length	0	N/A	N/A
>0 to ≤4 months	729	865	1 (1 to 1)
>4 to ≤9 months	1,133	1,353	1 (1 to 1)
>9 months to ≤2 years	1,306	3,254	2 (2 to 3)
Weight models:			
Overall	1,390	11,411	7 (6 to 9)
Birth weight	1,390	1,390	1
>0 to ≤4 months	1,255	3,549	2 (2 to 3)
>4 to ≤9 months	1,235	2,577	2 (1 to 2
>9 months to ≤2 years	1,325	3,895	3 (2 to 4)
Generation XXI, N=5,282			
Length/height models:			
Overall	5,282	86,936	9 (5 to 14)
Birth length	4,983	4,983	1
>0 to ≤3 months	5,220	17,337	3 (2 to 4)
>3 months to ≤1 year	5,227	28,045	3 (2 to 5)
>1 year to ≤3 years	5,175	26,898	3 (2 to 5)
>3 years to ≤6 years	4,386	9,673	2 (2 to 3)
Weight models:			
Overall	5,282	101,723	10 (5 to 16)
Birth weight	5,014	5,014	1
>0 to ≤10 days	2,508	3,049	1 (1 to 2)
>10 days to ≤3 months	5,220	23,949	3 (2 to 5)
>3 months to ≤1 year	5,227	31,439	4 (2 to 6)
>1 year to ≤3 years	5,175	28,268	3 (2 to 5)
>3 years to ≤6 years	4,386	10,004	2 (1 to 3)

	Number of children with at least one measurement per growth period	Total number of measurements	Median (IQR) of measurements per child*
Pelotas 2004, N=4,188			
Length/height models:			
Overall	4,188	19,721	5 (5 to 5)
Birth	4,188	4,188	1
3 month follow-up	3,979	3,979	1
1 year follow-up	3,903	3,903	1
2 year follow-up	3,857	3,857	1
4 year follow-up	3,794	3,794	1
Weight models:			
Overall	4,188	19,688	5 (5 to 5)
Birth	4,188	4,188	1
3 month follow-up	3,975	3,975	1
1 year follow-up	3,877	3,877	1
2 year follow-up	3,852	3,852	1
4 year follow-up	3,997	3,997	1
Probit, N=16,368			
Length/height models:			
Overall	16,368	139,427	9 (7 to 10)
Birth length	19,095	16,095	1
>0 to ≤3 months	16,207	35,387	2 (2 to 2)
>3 months to ≤1 year	16,152	48,036	3 (3 to 3)
>1 year to ≤5 years	13,958	39,909	3 (1 to 4)
Weight models:			
Overall	16,368	140,207	9 (7 to 11)
Birth weight	16,095	16,095	1
>0 to ≤3 months	16,208	35,418	2 (2 to 2)
>3 months to ≤1 year	16,154	48,083	3 (3 to 3)
>1 year to ≤5 years	13,972	40,611	3 (1 to 4)

* Where no IQR is given, this is because growth periods were defined between each follow-up, so each child has a maximum of one growth measurement per follow-up

Supplementary Table 3: Differences between observed measurements and those predicted by the multilevel model for Born in Bradford, Generation XXI, Pelotas 2004 and PROBIT

	Mean actual measurement (SD)	Mean predicted measurement (SD)	Mean difference (SD)
Born in Bradford, N=1,390			
Length/height models:			
Birth length	N/A	N/A	N/A
>0 to ≤4 months	51.32 (3.47)	54.51 (4.25)	-0.05 (1.28)
>4 to ≤9 months	69.24 (3.18)	68.57 (3.25)	0.05 (1.27)
>9 months to ≤2 years	80.14 (5.75)	79.85 (5.67)	-0.01 (1.42)
Weight models:			
Birth weight	3.21 (0.55)	3.19 (0.52)	0.02 (0.14)
>0 to ≤4 months	4.49 (1.12)	4.49 (1.08)	-0.002 (0.15)
>4 to ≤9 months	7.82 (1.19)	7.83 (1.16)	-0.01 (0.17)
>9 months to ≤2 years	10.67 (1.83)	10.68 (1.79)	0.001 (0.28)
Generation XXI, N=5,282			
Length/height models:			
Birth length	48.766 (2.311)	48.659 (1.912)	0.108 (1.153)
>0 to ≤3 months	53.970 (3.978)	54.135 (3.641)	-0.165 (1.649)
>3 months to ≤1 year	67.042 (5.172)	66.909 (4.936)	0.133 (1.498)
>1 year to ≤3 years	83.653 (7.153)	93.633 (6.612)	0.0198 (2.051)
>3 years to ≤6 years	100.906 (6.233)	101.174 (5.077)	-0.267 (2.698)
Weight models:			
Birth weight	3.19 (0.50)	3.16 (0.41)	0.023 (0.346)
>0 to ≤10 days	3.16 (0.56)	3.27 (0.44)	-0.110 (0.339)
>10 days to ≤3 months	4.32 (1.00)	4.28 (0.88)	0.031 (0.311)
>3 months to ≤1 year	7.72 (1.51)	7.73 (1.44)	-0.008 (0.338)
>1 year to ≤3 years	11.86 (2.14)	11.86 (2.02)	-0.003 (0.557)
>3 years to ≤6 years	16.64 (2.92)	16.67 (2.66)	-0.029 (0.784)
Pelotas 2004, N=4,188			
Length/height models:			
Birth	48.18 (2.60)	48.17 (1.97)	-0.0018 (1.3645)
3 month follow-up	59.92 (2.77)	59.98 (2.30)	0.0016 (1.5147)
1 year follow-up	74.28 (3.03)	74.43 (2.64)	-0.0001 (1.6057)
2 year follow-up	86.60 (3.66)	86.72 (3.34)	-0.0019 (1.6341)
4 year follow-up	103.29 (4.69)	102.23 (4.13)	0.002 (1.0950)
Weight models:			
Birth	3.17 (0.54)	3.17 (0.36)	-0.0001 (0.31)
3 month follow-up	5.78 (0.86)	5.78 (0.73)	-0.00002 (0.24)
1 year follow-up	9.15 (1.34)	9.13 (1.19)	0.02 (0.29)
2 year follow-up	11.78 (1.76)	11.85 (1.62)	-0.07 (0.55)
4 year follow-up	17.72 (3.24)	17.65 (2.78)	0.07 (0.83)
Probit, N=17,046			
Length/height models:			

Birth length	51.92 (2.13)	51.74 (1.76)	0.18 (0.67)
>0 to ≤3 months	56.79 (2.95)	56.99 (2.73)	-0.20 (0.80)
>3 months to ≤1 year	68.14 (5.51)	67.98 (5.38)	0.17 (1.39)
>1 year to ≤5 years	89.72 (11.70)	90.03 (11.37)	-0.24 (2.97)
Weight models:			
Birth weight	3.43 (0.42)	3.40 (0.33)	0.04 (0.18)
>0 to ≤3 months	4.91 (0.80)	4.97 (0.76)	-0.05 (0.20)
>3 months to ≤1 year	8.34 (1.71)	8.27 (1.66)	0.07 (0.40)
>1 year to ≤5 years	13.58 (3.02)	13.64 (2.62)	-0.06 (0.86)

Supplementary Table 4: Growth rates predicted by linear spline multilevel models for boys

Mean (SD) growth rates	ALSPAC	Born in Bradford (white British ethnicity)	Born in Bradford (Pakistani ethnicity)	Generation XXI	Pelotas 2004	PROBIT
Length/Height						
Boys, N	*7191*	*297*	*381*	*2518*	*2170*	*8463*
Birth length, cm	50.73 (1.69)	48.83 (1.34)	48.34 (1.14)	49.00 (1.94)	48.54 (2.00)	52.04 (1.79)
Early infancy, cm/month	3.86 (0.17)	3.89 (0.35)	4.19 (0.37)	4.12 (0.22)	4.04 (0.33)	3.16 (0.47)
Late infancy, cm/month	1.65 (0.15)	1.61 (0.29)	1.54 (0.25)	1.70 (0.18)	1.62 (0.15)	1.78 (0.18)
Early childhood, cm/month	0.80 (0.07)	0.93 (0.05)	0.94 (0.05)	0.88 (0.07)	1.01 (0.12)	0.74 (0.04)
Later childhood, cm/month	0.53 (0.04)	N/A	N/A	0.51 (0.02)	0.64 (0.05)	N/A
Weight						
Boys, N	*7191*	*297*	*381*	*2666*	*2170*	*8463*
Birth weight, kg	3.41 (0.50)	3.30 (0.57)	3.09 (0.50)	3.22 (0.43)	3.22 (0.37)	3.46 (0.33)
Early neonatal, kg /month	N/A	N/A	N/A	0.52 (0.70)	N/A	N/A
Early infancy, kg /month	1.04 (0.17)	0.97 (0.17)	0.97 (0.17)	0.99 (0.17)	0.94 (0.12)	0.96 (0.12)
Late infancy, kg /month	0.47 (0.09)	0.42 (0.12)	0.40 (0.13)	0.41 (0.09)	0.38 (0.08)	0.54 (0.08)
Early childhood, kg /month	0.18 (0.03)	0.22 (0.04)	0.24 (0.05)	0.22 (0.05)	0.22 (0.05)	0.15 (0.02)
Later childhood, kg /month	0.30 (0.10)	N/A	N/A	0.18 (0.05)	N/A	N/A

*Growth rates for early neonatal, early infancy, late infancy, early childhood and late childhood respectively: ALSPAC length/height: n/a, 0-3mt, 3-12mt, 1-3yrs, 3-10yrs, ALSPAC weight: n/a, 0-3mt, 3-12mt, 1-7 yrs, 7-10 yrs, BiB length and weight: n/a, 0-4mts, 4-9mts, 9-24mts, n/a, Generation XXI length/height: n/a, 0-3mts, 3-12mt, 1-3yrs, 3-6yrs, Generation XXI weight: 0-10days, 10days-3mts, 3-12mt, 1-3yrs, 3-6yrs, Pelotas length/height: n/a, 0-3mts, 3-12mts, 1-2yrs, 2-4yrs, Pelotas weight: n/a, 0-3mts, 3-24mts, 2-4yrs, n/a, PROBIT length/height and weight: n/a, 0-3mts, 3-12mts, 1-3yrs, 3-5yrs

Supplementary Table 5: Variance/covariance matrices between individual-level random effects

a1. ALSPAC weight trajectories

	Birth weight	Growth 0-3 months	Growth 3-12 months	Growth 1-7 years	Growth 7-10 years
**Birth weight**	0.19				
**Growth 0-3 months**	0.02	0.03			
**Growth 3-12 months**	-0.006	0.001	0.01		
**Growth 1-7 years**	0.004	0.0008	0.0003	0.002	
**Growth 7-10 years**	0.007	0.007	-0.0002	0.003	0.02

a2. ALSPAC length/height trajectories

	Birth length	Growth 0-3 months	Growth 3-12 months	Growth 1-3 years	Growth 3-10 years
**Birth length**	3.72				
**Growth 0-3 months**	0.12	0.16			
**Growth 3-12 months**	-0.17	-0.01	0.05		
**Growth 1-3 years**	0.02	-0.001	0.001	0.008	
**Growth 3-10 years**	0.02	0.004	0.0008	0.002	0.002

b1. BiB weight trajectories

	Birth weight	Growth 0-4 months	Growth 4-9 months	Growth 9 months – 2 years
**Birth weight**	0.29			
**Growth 0-4 months**	-0.0004	0.03		
**Growth 4-9 months**	-0.01	-0.002	0.02	
**Growth 9 mts – 2 yrs**	0.006	0.001	0.0005	0.003

b2. BiB length/height trajectories

	Birth length	Growth 0-4 months	Growth 4-9 months	Growth 9 months – 2 years
**Birth length**	3.56			
**Growth 0-4 months**	*	0.28		
**Growth 4-9 months**	-0.37	-0.08	0.18	
**Growth 9 mts – 2 yrs**	0.01	0.02	-0.005	0.008

*not estimated

c1. Generation XXI weight trajectories

	Birth weight	Growth 0-10 days	Growth 10 days – 3 months	Growth 3-12 months	Growth 1-3 years	Growth 3-6 years
**Birth weight**	0.21					
**Growth 0-10 days**	*	0.54				
**Growth 10 days – 3 mts**	0.006	-0.07	0.04			
**Growth 3-12 months**	0.003	-0.04	0.004	0.009		
**Growth 1-3 years**	0.005	0.003	0.0009	0.0009	0.003	
**Growth 3-6 years**	0.002	-0.009	0.004	0.001	0.002	0.01

*not estimated

c2. Generation XXI length/height trajectories

	Birth length	Growth 0-3 months	Growth 3-12 months	Growth 1-3 years	Growth 3-6 years
**Birth length**	4.26				
**Growth 0-3 months**	-0.008	0.12			
**Growth 3-12 months**	-0.17	0.01	0.04		
**Growth 1-3 years**	0.03	-0.003	0.004	0.01	
**Growth 3-6 years**	-0.03	0.01	-0.001	-0.0004	0.006

d1. Pelotas weight trajectories

	Birth weight	Growth 0-3 months	Growth 3-12 months	Growth 1-4 years
**Birth weight**	0.16			
**Growth 0-3 months**	0.04	0.02		
**Growth 3-12 months**	-0.0004	0.004	0.009	
**Growth 1-4 years**	0.005	0.002	0.002	0.003

d2. Pelotas length/height trajectories

	Birth length	Growth 0-3 months	Growth 3-12 months	Growth 1-2 years	Growth 2-4 years
**Birth length**	5.11				
**Growth 0-3 months**	*	0.06			
**Growth 3-12 months**	-0.14	0.03	0.03		
**Growth 1-2 years**	0.01	0.01	0.01	0.001	
**Growth 2-4 years**	0.004	0.008	0.003	0.008	0.0008

*not estimated

e1. PROBIT weight trajectories

	Birth weight	Growth 0-3 months	Growth 3-12 months	Growth 1-5 years
**Birth weight**	0.15			
**Growth 0-3 months**	0.003	0.02		
**Growth 3-12 months**	-0.006	0.001	0.007	
**Growth 1-5 years**	0.003	-0.0001	0.0006	0.002

e2. PROBIT length/height trajectories

	Birth length	Growth 0-3 months	Growth 3-12 months	Growth 1-5 years
**Birth length**	3.59			
**Growth 0-3 months**	-0.34	0.34		
**Growth 3-12 months**	-0.16	-0.00003	0.05	
**Growth 1-5 years**	0.004	-0.0003	0.001	0.007

Reference List

(1) Raynor P. Born in Bradford, a cohort study of babies born in Bradford, and their parents: protocol for the recruitment phase. BMC Public Health 2008;8:327.

(2) Kramer MS, Chalmers B, Hodnett ED, Sevkovskaya Z, Dzikovich I, Shapiro S, et al. Promotion of Breastfeeding Intervention Trial (PROBIT): a randomized trial in the Republic of Belarus. Jama 2001 Jan 24;285(4):413-20.

(3) Howe LD, Tilling K, Lawlor DA. Accuracy of height and weight data from child health records. Archives of Disease in Childhood 2009;94:950-4.

(4) Fraser A, Tilling K, Macdonald-Wallis C, Sattar N, Brion MJ, Benfield L, et al. Association of maternal weight gain in pregnancy with offspring obesity and metabolic and vascular traits in childhood. Circulation 2010 Jun 15;121(23):2557-64.
